# longmixr: a tool for robust clustering of high-dimensional cross-sectional and longitudinal variables of mixed data types

**DOI:** 10.1093/bioinformatics/btae137

**Published:** 2024-03-14

**Authors:** Jonas Hagenberg, Monika Budde, Teodora Pandeva, Ivan Kondofersky, Sabrina K Schaupp, Fabian J Theis, Thomas G Schulze, Nikola S Müller, Urs Heilbronner, Richa Batra, Janine Knauer-Arloth

**Affiliations:** Max Planck Institute of Psychiatry, 80804 Munich, Germany; International Max Planck Research School for Translational Psychiatry, 80804 Munich, Germany; Institute of Computational Biology, Helmholtz Zentrum München, 85764 Neuherberg, Germany; Institute of Psychiatric Phenomics and Genomics (IPPG), LMU University Hospital, LMU Munich, 80336 Munich, Germany; Institute of Computational Biology, Helmholtz Zentrum München, 85764 Neuherberg, Germany; AI4Science, AMLab, University of Amsterdam, GH 1090 Amsterdam, The Netherlands; Swammerdam Institute for Life Sciences, University of Amsterdam, GE 1090 Amsterdam, The Netherlands; Institute of Computational Biology, Helmholtz Zentrum München, 85764 Neuherberg, Germany; Institute of Psychiatric Phenomics and Genomics (IPPG), LMU University Hospital, LMU Munich, 80336 Munich, Germany; Institute of Computational Biology, Helmholtz Zentrum München, 85764 Neuherberg, Germany; Department of Mathematics, Technical University of Munich, 85748 Munich, Germany; Institute of Psychiatric Phenomics and Genomics (IPPG), LMU University Hospital, LMU Munich, 80336 Munich, Germany; Department of Psychiatry and Behavioral Sciences, SUNY Upstate Medical University, Syracuse, NY 13210, United States; Department of Psychiatry and Behavioral Sciences, Johns Hopkins University School of Medicine, Baltimore, MD 21287, United States; Institute of Computational Biology, Helmholtz Zentrum München, 85764 Neuherberg, Germany; Institute of Psychiatric Phenomics and Genomics (IPPG), LMU University Hospital, LMU Munich, 80336 Munich, Germany; Institute of Computational Biology, Helmholtz Zentrum München, 85764 Neuherberg, Germany; Institute for Computational Biomedicine, Weill Cornell Medical College of Cornell University, New York, NY 10021, United States; Max Planck Institute of Psychiatry, 80804 Munich, Germany; Institute of Computational Biology, Helmholtz Zentrum München, 85764 Neuherberg, Germany

## Abstract

**Summary:**

Accurate clustering of mixed data, encompassing binary, categorical, and continuous variables, is vital for effective patient stratification in clinical questionnaire analysis. To address this need, we present longmixr, a comprehensive R package providing a robust framework for clustering mixed longitudinal data using finite mixture modeling techniques. By incorporating consensus clustering, longmixr ensures reliable and stable clustering results. Moreover, the package includes a detailed vignette that facilitates cluster exploration and visualization.

**Availability and implementation:**

The R package is freely available at https://cran.r-project.org/package=longmixr with detailed documentation, including a case vignette, at https://cellmapslab.github.io/longmixr/.

## 1 Introduction

Identifying groups of individuals that show similar trajectories on a number of distinct clinical measures is of great interest to healthcare researchers. Identifying such groups is especially critical in the field of mental health research, where questionnaires and rating scales are ubiquitous and laboratory-based measurements play a smaller role. Moreover, different psychiatric disorders often share similar features, increasingly challenging the traditional categorical classification of mental illness. For example, studies have shown that schizophrenia and bipolar disorder exhibit significant phenotypic and genetic overlap and share responses to the same class of medications (David *et al.* 2023). Alternative dimensional frameworks have thus been proposed (Insel *et al.* 2010, Kotov *et al.* 2017), but the important question of how to identify meaningful groups of individuals with similar trajectories remains (Weinberger *et al.* 2015).

To this end, we suggested that simultaneously examining the longitudinal course of psychiatric rating scales, cognitive tests and questionnaires appears to be a promising approach (Budde *et al.* 2019). Over the years, various methodological approaches for analyzing longitudinal trajectories have emerged, as highlighted by Den Teuling *et al.* 2023 and Lu *et al.* 2023. Among these, several R packages facilitate longitudinal clustering, including those based on functional data analysis, such as CONNECTOR (Pernice *et al.* 2023), k-means clustering (kml3d, Genolini *et al.* 2013), or model-based clustering (longclust, McNicholas and Murphy 2010). A major challenge in analyzing large clinical datasets, not addressed by the aforementioned methods, is the mixed data types (e.g. binary, categorical, or continuous) of clinical phenotypes. While the model-based clustering packages lcmm (Proust-Lima *et al.* 2017), mixAK (Komárek and Komárková 2014) and BCClong (Tan, Lu and Shen 2023) can cluster longitudinal mixed data, they do not offer feature reduction nor repeatedly cluster on subsets of the data for a robust selection of the number of clusters.

To address this critical gap, we present longmixr, an innovative R package for analyzing longitudinal phenotype data with mixed data types. It offers a robust model-based clustering method and a detailed vignette for effective data visualization and exploration, enabling the identification of meaningful subgroups of individuals. Longmixr also includes instructions on dimensionality reduction techniques for large clinical datasets and features to cluster multivariate longitudinal mixed data repeatedly on subsets for robustness, thus providing a robust end-to-end pipeline for mixed data.

## 2 Clustering strategy

**Figure 1.**
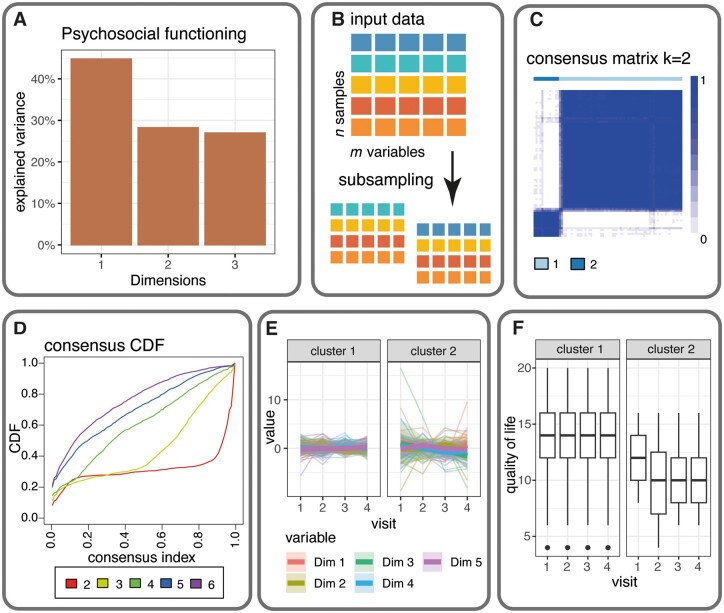
The longmixr workflow for longitudinal data. (A) Longmixr can deal with categorical and continuous input data, its dimensions are reduced by Factor Analysis of Mixed Data (FAMD). (B) For the consensus clustering, the input data gets subsampled and the clustering is applied to every subsample. Longmixr uses a model-based clustering using the R package flexmix (Grün and Leisch 2008). The determination of the optimal number of clusters is based on the consensus matrix and the consensus cumulative distribution function (CDF). (C) Heatmap of the consensus matrix for a two cluster solution; the darker the color the more often two individuals are clustered together in the same cluster. (D) The consensus CDF plot for different solutions with 2–6 clusters. (E) Time course visualization of the variables across the identified clusters. (F) The global quality of life measured by the WHOQOL-BREF questionnaire across the identified clusters that was not used in the clustering.

In the following sections, we present a workflow to robustly cluster mixed data (Fig. 1). The clustering is based on consensus clustering that performs repeated clusterings on different subsets of the data, combines the results in a consensus matrix, and performs a final hierarchical clustering step on this matrix (Monti *et al.* 2003). Notably, the workflow of longmixr assumes a complete dataset. In case of missing data, users can utilize imputation techniques to address this issue (van Buuren 2018).

### 2.1 Consensus clustering for longitudinal data

Compared to cross-sectional data, longitudinal data poses an additional challenge due to the presence of repeated measurements from the same individual, introducing a lack of independence among observations. To reflect this in the clustering algorithm, the R package flexmix is used for the clustering step on subsets of the data (Grün and Leisch 2008). The rest of the consensus clustering is kept consistent with the approach described above, and the implementation is based upon the R package ConsensusClusterPlus (Wilkerson and Hayes 2010).

Flexmix iteratively fits a mixture of generalized additive models for a different number of components. All observations are then assigned to one of the components which can be regarded as clusters. Flexmix has the built-in functionality to deal with repeated measurements. The observed variables are modeled using a thin plate regression spline smoothing function for the time points, with the sample IDs grouping the repeated measurements. The spline capabilities are provided by the FLXMRmgcv adapter.

For every response variable, a different flexmix model is generated and all models are estimated simultaneously, i.e. interpreting all observed variables as a multivariate outcome. A caveat to be aware of is that flexmix assumes that the variables are independent. We partially address this requirement by using the components from a dimension reduction algorithm, which are designed to be orthogonal.

This workflow is implemented in the function longitudinal_consensus_cluster. The longmixr R package offers a default of 100 repetitions to manage computational costs. However, it provides users with the flexibility to utilize the complete range of flexmix models for clustering, allowing for customization based on individual requirements.

### 2.2 Dimension reduction

As clinical datasets can be large with tens of questionnaires and thousands to hundred thousands subjects assessed at several time points (Northstone *et al.* 2019, Penninx *et al.* 2021, Peters *et al.* 2022), and for each variable a model will be created during the clustering process for longitudinal data, thus it is crucial to reduce the dimensionality of the dataset. We use the function FAMD (Factor Analysis of Mixed Data) from the R package FactoMineR (Lê *et al.* 2008) as it is a principal component method that can deal with both continuous and categorical data. The individual factor scores of the different dimensions are then used as the dependent variables for the identification of clusters with flexmix. Dimensionality reduction is performed separately for specific measurement groupings when phenotypic measurements can be meaningfully divided based on prior knowledge (as shown in the section Application of longmixr on real data). This approach improves result interpretation by focusing on each grouping's reduced dimensionality, enabling a clearer understanding of the clustering outcomes. Alternatively, it is possible to perform a completely unsupervised dimension reduction without the previous grouping.

### 2.3 Diagnostic plots to determine the number of clusters

To determine the optimal number of clusters, longmixr provides the same diagnostic plots available in the ConsensusClusterPlus package. We recommend the visualization of the consensus matrices, the consensus cumulative distribution function (CDF) plot and the item-consensus plots to determine the optimal number of clusters. The plots can be produced by calling the plot function on an lcc object generated by the longmixr package. The interpretation of these plots is discussed in Monti *et al.* (2003). In short, a good cluster solution should show a clear separation of the clusters in the consensus matrix and its line in the consensus CDF plot should show a binary separation with a steep ascent at 0, then flat and another steep ascent toward 1.

### 2.4 Consensus clustering for cross-sectional data

The package also contains the function crosssectional_consensus_cluster that provides a wrapper to the ConsensusClusterPlus package with sensible defaults for cross-sectional data with mixed data types. See Supplementary section Consensus clustering for cross-sectional categorical data for more details.

## 3 Application of longmixr on simulated data

To assess the performance of longmixr, we conducted a simulation study where we simulated longitudinal mixed data resembling questionnaire data with two to four groups for 50, 100, 200, 500, and 1000 individuals. The rand index for two clusters was 1 for all numbers of individuals, for three clusters 0.947–0.977 and for four clusters 0.944–0.985 (Supplementary Table S1). Two clusters can be reliably detected already with 50 observations by assessing the consensus CDF plot. To unambiguously detect three clusters, at least 100 individuals are needed. While the rand index for four groups shows a good overlap with the four clustering solution, even for the big sample sizes the consensus CDF plot suggests a five cluster solution. On the other hand, the consensus matrix plots are ambiguous between a three or four cluster solution but do not support a five cluster solution (Supplementary Figs S1–S6). This shows the complexities of deciding which number of clusters suits the data and underscores the need to jointly assess several diagnostic plots as provided by longmixr (see the Supplementary section Application of longmixr on simulated data).

## 4 Application of longmixr on real data

In addition, we applied longmixr to a real dataset. The data used to illustrate the longitudinal part of longmixr are 76 participants diagnosed with schizophrenia according to the Diagnostic and Statistical Manual of Mental Disorders, Fourth Edition (DSM-IV) from the transdiagnostic psychiatric PsyCourse Study (PMID: 30070057, Budde *et al.* 2019) and was obtained from Heilbronner *et al.* (2023). Different rating scales and questionnaires from these samples were used for the clustering. The variables were grouped based on prior knowledge (groups based on specific rating scales and semantic relatedness, e.g. a functioning domain). FAMD was applied per group of variables to reduce the data dimensionality (Fig. 1A). In addition, age as a covariate was regressed out of the components (Supplementary Fig. S7). Based on the consensus matrices, the consensus CDF and the item-consensus plots (Fig. 1C and D and Supplementary Figs S8–S15), we determined two clusters as the optimal cluster solution. One cluster contains patients with more severe symptoms and a higher variability over time compared to the other cluster (Fig. 1E and Supplementary Figs S16–S23). The difference in symptom severity can also be observed in the quality of life questionnaire (WHOQOL-BREF) not used in the clustering (Fig. 1F and Supplementary Table S2), indicating a stable clustering result. For the detailed workflow, see the Supplementary section Application of longmixr on real data and the vignette of the longmixr package.

## 5 Conclusion

The methodology presented in the longmixr package has wide application in the biomedical field beyond neuropsychiatry. By combining longitudinal modeling with consensus clustering, it facilitates robust analyses in various fields that utilize questionnaire data. Moreover, the flexibility of flexmix allows for the incorporation of more complex data modeling approaches, expanding the potential applications beyond the scope of the current paper.

## Supplementary Material

btae137_Supplementary_Data

## Data Availability

No new data were generated or analysed in support of this research.
