# Supplementary material for: longmixr: a tool for robust clustering of high-dimensional cross-sectional and longitudinal variables of mixed data types
Source: Bioinformatics. 2024 Mar 14;40(4):btae137. doi: 10.1093/bioinformatics/btae137 (PMC10994717; doi:10.1093/bioinformatics/btae137)
Supplement: btae137_Supplementary_Data [file btae137_supplementary_data.zip › supplementary_simulation_code_longmixr_Hagenberg.pdf]

Simulation study code for ‘longmixr: A tool for robust clustering of high-dimensional cross-sectional and longitudinal phenotype data’

Jonas Hagenberg Monika Budde Teodora Pandeva Ivan Kondofersky
Sabrina K. Schaupp Fabian J. Theis Thomas G. Schulze Nikola S. Müller
Urs Heilbronner Richa Batra Janine Knauer-Arloth

Contents

1 Data simulation **1**
1.1 Mixed model . . . 1
1.2 IRT for categorical data . . . 12
1.3 Create one data source . . . 18

2 Run the simulation **20**

```
library(dplyr)
library(catIrt)
library(purrr)
```

1 Data simulation

The data is simulated in two steps:

1. generate a continuous variable from a mixed model
2. use this variable as theta (latent variable or ability) in a graded response model (GRM)

The theta in the GRM should follow a N(0, 1) distribution. Therefore, the values from step 1 are mapped to the quantiles of a N(0, 1) function. For every questionnaire, one mixed model is used. In the GRM, the number of the items per questionnaire, the discrimination of each item and the difficulty/ threshold for every class (the value of theta at which the probability to select the next class is 50%) can be varied.

In total, 50, 100, 200, 500 and 1000 individuals aged between 18 - 65 with 4 time points in 4 equally sized groups are simulated with 3 questionnaires:

- 15 items with 5 levels each
- 20 items with 4 levels each
- 2 items with 5 levels each and 3 continuous variables

Additionally, a cross-sectional continuous variable is simulated that could be used to compare the groups after clustering.

1.1 Mixed model

```
set.seed(87)

# simulate the age
```

```r
age_df <- data.frame(ID = 1:1000,
                     age_visit_1 = round(runif(200, min = 18, max = 65)))
```

```r
# adapted from
# https://stats.stackexchange.com/questions/394092/simulate-longitudinal-curvilinear-convergent-data-in

simulate_longi <- function(
    n = 200,
    n_timepoints = 4,
    n_groups = 2,
    x_formula = formula(~ group * (visit + I(visit^2)) + age_visit_1),
    z_formula = formula(~ (visit + I(visit^2))),
    betas = c(23, 25, 1.74, 0.033, 0.1, 0, -4),
    random_var = c(D11 = 0.0001, D22 = 0.0001, D33 = 0.0001),
    sigma = 1,
    age_info = age_df
) {
  # create general data.frame with the repeated IDs for the visits and
  # to which group they belong
  data <- data.frame(ID = rep(seq_len(n), each = n_timepoints),
                     visit = rep(1:4, n),
                     group = rep(gl(n_groups, n / n_groups,
                                    labels = LETTERS[seq_len(n_groups)]),
                                 each = n_timepoints))

  if (!is.null(age_info)) {
    data <- dplyr::left_join(data, age_info, by = "ID")
  }

  # design matrices for the fixed and random effects
  X_matrix <- model.matrix(x_formula, data = data)
  Z_matrix <- model.matrix(z_formula, data = data)

  # simulate random effects
  # this creates a matrix with a random effect for every individual and
  # variable that is contained in the z_formula
  b <- lapply(random_var, function(d) {
    rnorm(n, sd = sqrt(d))
  })
  b <- do.call(cbind, b)

  # calculate the linear predictor
  # the random effects are repeated per individual for every time point and
  # summed up
  eta <- drop(X_matrix %*% betas + rowSums(Z_matrix * b[data$ID, ]))

  # simulate the longitudinal data
  data <- data %>%
    mutate(y = rnorm(n * n_timepoints, eta, sigma))

  # remove again the age because I add it later to the merged data
  if (!is.null(age_info)) {
    data <- data %>%
      select(-age_visit_1)
```

```
  }

  data
}
```

Normalisation function:

```
quantile_normalisation <- function(data) {
  # check for NAs
  data <- data.frame(value = data)
  index_use <- which(!is.na(data$value) &
                     !is.nan(data$value))
  if (length(index_use) == 0) {
    stop("No data provided")
  }

  # make the normalisation
  data$norm <- NA
  data[index_use, "norm"] <-
    qnorm(rank(data[index_use, "value"]) / (nrow(data[index_use, ]) + 1))

  # return the value
  data$norm
}
```

1.1.1 2 groups

```
group_2_latent_1 <- c(50, 100, 200, 500, 1000) %>%
  set_names() %>%
  map(~simulate_longi(
    n = .x,
    n_groups = 2,
    # intercept, groupB, visit, visit^2,
    # age_visit_1, groupB:visit, groupB:visit^2
    betas = c(-0.5, 3, 1.3, 0.025, 0.1, 0.5, -2)
  ))

# normalise the data
group_2_latent_1 <- group_2_latent_1 %>%
  map(function(data) {
    data %>%
      mutate(y_norm = quantile_normalisation(y))
  })
```

```
group_2_latent_2 <- c(50, 100, 200, 500, 1000) %>%
  set_names() %>%
  map(~simulate_longi(
    n = .x,
    n_groups = 2,
    # intercept, groupB, visit, visit^2,
    # visit^3, age_visit_1, groupB:visit
    # groupB:visit^2, groupB:visit^3
    betas = c(0, 3, -5, 1,
              0, 0.1, 23.75,
```

```
              -8.5, 0.9),
    random_var = c(D11 = 0.0001),
    x_formula = formula(~ group * (visit + I(visit^2) + I(visit^3)) + age_visit_1),
    z_formula = formula(~ 1)
  ))

# normalise the data
group_2_latent_2 <- group_2_latent_2 %>%
  map(function(data) {
    data %>%
      mutate(y_norm = quantile_normalisation(y))
  })
```

The third questionnaire consists of both categorical variables and continuous variables. The continuous variables are directly modeled with the mixed model.

```
group_2_latent_3 <- c(50, 100, 200, 500, 1000) %>%
  set_names() %>%
  map(~simulate_longi(
    n = .x,
    n_groups = 2,
    # intercept, groupB, visit,
    # age_visit_1, groupB:visit
    betas = c(0, 6.5, 0.8,
              -0.2, 0),
    random_var = c(D11 = 0.0001),
    x_formula = formula(~ group * (visit) + age_visit_1),
    z_formula = formula(~ 1),
    sigma = 0.5
  ))

# normalise the data
group_2_latent_3 <- group_2_latent_3 %>%
  map(function(data) {
    data %>%
      mutate(y_norm = quantile_normalisation(y))
  })
```

```
group_2_continuous_3_1 <- c(50, 100, 200, 500, 1000) %>%
  set_names() %>%
  map(~simulate_longi(
    n = .x,
    n_groups = 2,
    # intercept, groupB, visit,
    # age_visit_1, groupB:visit
    betas = c(-1, 7, 1.2,
              0, 0.5),
    random_var = c(D11 = 0.0001),
    x_formula = formula(~ group * (visit) + age_visit_1),
    z_formula = formula(~ 1),
    sigma = 1
  ))

# normalise the data
```

```r
group_2_continuous_3_1 <- group_2_continuous_3_1 %>%
  map(function(data) {
    data %>%
      mutate(y_norm = quantile_normalisation(y))
  })
```

```r
group_2_continuous_3_2 <- c(50, 100, 200, 500, 1000) %>%
  set_names() %>%
  map(~simulate_longi(
    n = .x,
    n_groups = 2,
    # intercept, groupB, visit,
    # age_visit_1, groupB:visit
    betas = c(3, 0, 0,
              -0.05, 0),
    random_var = c(D11 = 0.0001),
    x_formula = formula(~ group * (visit) + age_visit_1),
    z_formula = formula(~ 1),
    sigma = 1
  ))

# normalise the data
group_2_continuous_3_2 <- group_2_continuous_3_2 %>%
  map(function(data) {
    data %>%
      mutate(y_norm = quantile_normalisation(y))
  })
```

```r
group_2_continuous_3_3 <- c(50, 100, 200, 500, 1000) %>%
  set_names() %>%
  map(~simulate_longi(
    n = .x,
    n_groups = 2,
    # intercept, groupB, visit, visit^2,
    # visit^3, age_visit_1, groupB:visit
    # groupB:visit^2
    # groupB:visit^3
    betas = c(0, 0, 4, -0.5,
              0, 0.1, -1,
              -2,
              0.7),
    random_var = c(D11 = 0.0001),
    x_formula = formula(~ group * (visit + I(visit^2) + I(visit^3)) + age_visit_1),
    z_formula = formula(~ 1),
    sigma = 1
  ))

# normalise the data
group_2_continuous_3_3 <- group_2_continuous_3_3 %>%
  map(function(data) {
    data %>%
      mutate(y_norm = quantile_normalisation(y))
  })
```

Create one continuous cross-sectional variable:

```
group_2_continuous_cr <- c(50, 100, 200, 500, 1000) %>%
  set_names() %>%
  map(~simulate_longi(
    n = .x,
    n_groups = 2,
    # intercept, groupB, age_visit_1
    betas = c(3, -2, 0.05),
    random_var = c(D11 = 0.0001),
    x_formula = formula(~ group + age_visit_1),
    z_formula = formula(~ 1),
    sigma = 1
  ))

# Only keep the first visit value
group_2_continuous_cr <- group_2_continuous_cr %>%
  map(function(data) {
    data %>%
      filter(visit == 1)
  })
```

1.1.2 3 groups

```
group_3_latent_1 <- c(50, 100, 200, 500, 1000) %>%
  set_names() %>%
  map(~simulate_longi(
    n = .x,
    n_groups = 3,
    # intercept, groupB, groupC, visit, visit^2,
    # age_visit_1, groupB:visit, groupC:visit
    # groupB:visit^2, groupC:visit^2
    betas = c(-0.5, 3, -4, 1.3, 0.025, 0.1, 0.5, 0, -2, 2)
  ))

# normalise the data
group_3_latent_1 <- group_3_latent_1 %>%
  map(function(data) {
    data %>%
      mutate(y_norm = quantile_normalisation(y))
  })
```

```
group_3_latent_2 <- c(50, 100, 200, 500, 1000) %>%
  set_names() %>%
  map(~simulate_longi(
    n = .x,
    n_groups = 3,
    # intercept, groupB, groupC, visit, visit^2,
    # visit^3, age_visit_1, groupB:visit, groupC:visit
    # groupB:visit^2, groupC:visit^2
    # groupB:visit^3, groupC:visit^3
    betas = c(-18.625, 3, 3, -5, 1,
              0, 0.1, 23.75, 3.5,
              -8.5, -1,
```

```
              0.9, 0),
    random_var = c(D11 = 0.0001),
    x_formula = formula(~ group * (visit + I(visit^2) + I(visit^3)) + age_visit_1),
    z_formula = formula(~ 1)
  ))

# normalise the data
group_3_latent_2 <- group_3_latent_2 %>%
  map(function(data) {
    data %>%
      mutate(y_norm = quantile_normalisation(y))
  })
```

The third questionnaire consists of both categorical variables and continuous variables. The continuous variables are directly modeled with the mixed model.

```
group_3_latent_3 <- c(50, 100, 200, 500, 1000) %>%
  set_names() %>%
  map(~simulate_longi(
    n = .x,
    n_groups = 3,
    # intercept, groupB, groupC, visit,
    # age_visit_1, groupB:visit, groupC:visit
    betas = c(0, 8, -2, 0.8,
              -0.2, 0, 0),
    random_var = c(D11 = 0.0001),
    x_formula = formula(~ group * (visit) + age_visit_1),
    z_formula = formula(~ 1),
    sigma = 0.5
  ))

# normalise the data
group_3_latent_3 <- group_3_latent_3 %>%
  map(function(data) {
    data %>%
      mutate(y_norm = quantile_normalisation(y))
  })
```

```
group_3_continuous_3_1 <- c(50, 100, 200, 500, 1000) %>%
  set_names() %>%
  map(~simulate_longi(
    n = .x,
    n_groups = 3,
    # intercept, groupB, groupC, visit,
    # age_visit_1, groupB:visit, groupC:visit
    betas = c(-1, 0, 7, 1.2,
              0, -3, 0.5),
    random_var = c(D11 = 0.0001),
    x_formula = formula(~ group * (visit) + age_visit_1),
    z_formula = formula(~ 1),
    sigma = 1
  ))

# normalise the data
```

```r
group_3_continuous_3_1 <- group_3_continuous_3_1 %>%
  map(function(data) {
    data %>%
      mutate(y_norm = quantile_normalisation(y))
  })
```

```r
group_3_continuous_3_2 <- c(50, 100, 200, 500, 1000) %>%
  set_names() %>%
  map(~simulate_longi(
    n = .x,
    n_groups = 3,
    # intercept, groupB, groupC, visit,
    # age_visit_1, groupB:visit, groupC:visit
    betas = c(3, 0, 0.1, 0,
              -0.05, 0, 0),
    random_var = c(D11 = 0.0001),
    x_formula = formula(~ group * (visit) + age_visit_1),
    z_formula = formula(~ 1),
    sigma = 1
  ))

# normalise the data
group_3_continuous_3_2 <- group_3_continuous_3_2 %>%
  map(function(data) {
    data %>%
      mutate(y_norm = quantile_normalisation(y))
  })
```

```r
group_3_continuous_3_3 <- c(50, 100, 200, 500, 1000) %>%
  set_names() %>%
  map(~simulate_longi(
    n = .x,
    n_groups = 3,
    # intercept, groupB, groupC, visit, visit^2,
    # visit^3, age_visit_1, groupB:visit, groupC:visit
    # groupB:visit^2, groupC:visit^2
    # groupB:visit^3, groupC:visit^3
    betas = c(0, -3, 0, 4, -0.5,
              0, 0.1, 3, -1,
              0, -2,
              0, 0.7),
    random_var = c(D11 = 0.0001),
    x_formula = formula(~ group * (visit + I(visit^2) + I(visit^3)) + age_visit_1),
    z_formula = formula(~ 1),
    sigma = 1
  ))

# normalise the data
group_3_continuous_3_3 <- group_3_continuous_3_3 %>%
  map(function(data) {
    data %>%
      mutate(y_norm = quantile_normalisation(y))
  })
```

Create one continuous cross-sectional variable:

```
group_3_continuous_cr <- c(50, 100, 200, 500, 1000) %>%
  set_names() %>%
  map(~simulate_longi(
    n = .x,
    n_groups = 3,
    # intercept, groupB, groupC, age_visit_1
    betas = c(3, -2, 2.5, 0.05),
    random_var = c(D11 = 0.0001),
    x_formula = formula(~ group + age_visit_1),
    z_formula = formula(~ 1),
    sigma = 1
  ))

# Only keep the first visit value
group_3_continuous_cr <- group_3_continuous_cr %>%
  map(function(data) {
    data %>%
      filter(visit == 1)
  })
```

1.1.3 4 groups

```
group_4_latent_1 <- c(50, 100, 200, 500, 1000) %>%
  set_names() %>%
  map(~simulate_longi(
    n = .x,
    n_groups = 4,
    # intercept, groupB, groupC, groupD, visit, visit^2,
    # age_visit_1, groupB:visit, groupC:visit, groupD:visit,
    # groupB:visit^2, groupC:visit^2, groupD:visit^2
    betas = c(-0.5, 2.5, 3, -4, 1.3, 0.025, 0.1, 1, 0.5, 0, 0.3, -2, 2)
  ))

# normalise the data
group_4_latent_1 <- group_4_latent_1 %>%
  map(function(data) {
    data %>%
      mutate(y_norm = quantile_normalisation(y))
  })
```

```
group_4_latent_2 <- c(50, 100, 200, 500, 1000) %>%
  set_names() %>%
  map(~simulate_longi(
    n = .x,
    n_groups = 4,
    # intercept, groupB, groupC, groupD, visit, visit^2,
    # visit^3, age_visit_1, groupB:visit, groupC:visit, groupD:visit,
    # groupB:visit^2, groupC:visit^2, groupD:visit^2,
    # groupB:visit^3, groupC:visit^3, groupD:visit^3
    betas = c(-18.625, 3, 3, 3, -5, 1,
              0, 0.1, 23.75, 3.5, 3.5,
              -8.5, -1, -1,
```

```
             0.9, 0, 0),
    random_var = c(D11 = 0.0001),
    x_formula = formula(~ group * (visit + I(visit^2) + I(visit^3)) + age_visit_1),
    z_formula = formula(~ 1)
  ))

# normalise the data
group_4_latent_2 <- group_4_latent_2 %>%
  map(function(data) {
    data %>%
      mutate(y_norm = quantile_normalisation(y))
  })
```

The third questionnaire consists of both categorical variables and continuous variables. The continuous variables are directly modeled with the mixed model.

```
group_4_latent_3 <- c(50, 100, 200, 500, 1000) %>%
  set_names() %>%
  map(~simulate_longi(
    n = .x,
    n_groups = 4,
    # intercept, groupB, groupC, groupD, visit,
    # age_visit_1, groupB:visit, groupC:visit, groupD:visit
    betas = c(0, 8, -2, 0, 0.8,
             -0.2, 0, 0, 0),
    random_var = c(D11 = 0.0001),
    x_formula = formula(~ group * (visit) + age_visit_1),
    z_formula = formula(~ 1),
    sigma = 0.5
  ))

# normalise the data
group_4_latent_3 <- group_4_latent_3 %>%
  map(function(data) {
    data %>%
      mutate(y_norm = quantile_normalisation(y))
  })
```

```
group_4_continuous_3_1 <- c(50, 100, 200, 500, 1000) %>%
  set_names() %>%
  map(~simulate_longi(
    n = .x,
    n_groups = 4,
    # intercept, groupB, groupC, groupD, visit,
    # age_visit_1, groupB:visit, groupC:visit, groupD:visit
    betas = c(-1, -3, 0, 7, 1.2,
             0, 0.1, -3, 0.5),
    random_var = c(D11 = 0.0001),
    x_formula = formula(~ group * (visit) + age_visit_1),
    z_formula = formula(~ 1),
    sigma = 1
  ))

# normalise the data
```

```r
group_4_continuous_3_1 <- group_4_continuous_3_1 %>%
  map(function(data) {
    data %>%
      mutate(y_norm = quantile_normalisation(y))
  })
```

```r
group_4_continuous_3_2 <- c(50, 100, 200, 500, 1000) %>%
  set_names() %>%
  map(~simulate_longi(
    n = .x,
    n_groups = 4,
    # intercept, groupB, groupC, groupD, visit,
    # age_visit_1, groupB:visit, groupC:visit, groupD:visit
    betas = c(3, 0, 0.1, -0.2, 0,
              -0.05, 0, 0, 0),
    random_var = c(D11 = 0.0001),
    x_formula = formula(~ group * (visit) + age_visit_1),
    z_formula = formula(~ 1),
    sigma = 1
  ))

# normalise the data
group_4_continuous_3_2 <- group_4_continuous_3_2 %>%
  map(function(data) {
    data %>%
      mutate(y_norm = quantile_normalisation(y))
  })
```

```r
group_4_continuous_3_3 <- c(50, 100, 200, 500, 1000) %>%
  set_names() %>%
  map(~simulate_longi(
    n = .x,
    n_groups = 4,
    # intercept, groupB, groupC, groupD, visit, visit^2,
    # visit^3, age_visit_1, groupB:visit, groupC:visit, groupD:visit,
    # groupB:visit^2, groupC:visit^2, groupD:visit^2,
    # groupB:visit^3, groupC:visit^3, groupD:visit^3
    betas = c(0, -2, 3, 0, 4, -0.5,
              0, 0.1, 1, 3, -1,
              0, 0, -2,
              0, 0, 0.7),
    random_var = c(D11 = 0.0001),
    x_formula = formula(~ group * (visit + I(visit^2) + I(visit^3)) + age_visit_1),
    z_formula = formula(~ 1),
    sigma = 1
  ))

# normalise the data
group_4_continuous_3_3 <- group_4_continuous_3_3 %>%
  map(function(data) {
    data %>%
      mutate(y_norm = quantile_normalisation(y))
  })
```

Create one continuous cross-sectional variable:

```
group_4_continuous_cr <- c(50, 100, 200, 500, 1000) %>%
  set_names() %>%
  map(~simulate_longi(
    n = .x,
    n_groups = 4,
    # intercept, groupB, groupC, groupD, age_visit_1
    betas = c(3, -2, 2.5, -1.3, 0.05),
    random_var = c(D11 = 0.0001),
    x_formula = formula(~ group + age_visit_1),
    z_formula = formula(~ 1),
    sigma = 1
  ))

# Only keep the first visit value
group_4_continuous_cr <- group_4_continuous_cr %>%
  map(function(data) {
    data %>%
      filter(visit == 1)
  })
```

1.2 IRT for categorical data

The a parameter gives the differentiation, the higher the value the better the differentiation between the different levels. The b parameters give the level boundaries, it is the theta value at which the probability is 50% that this or a higher level is selected.

Define the parameters for the second questionnaire:

```
set.seed(3755)

params_q2 <- cbind(
  a = c(0.5, 1, 1.5, 2, 2.5, 0.8, 1.9, 1.1, 0.6, 1.7, 0.83, 1.2, 1.8, 0.9,
        1.45, 0.4, 1, 1.79, 0.7, 1.5),
  b1 = runif(20, min = -3, max = -1.5),
  b2 = runif(20, min = -1.5, max = 0),
  b3 = runif(20, min = 0, max = 1.5)
)
```

1.2.1 2 groups

1.2.1.1 Questionnaire 1 Create questionnaire 1 with 15 items and 5 levels each:

```
group_2_irt_1 <- group_2_latent_1 %>%
  map(function(data) {
    simIrt(
      theta = data$y_norm,
      params = cbind(
        a = c(0.5, 1, 1.5, 1.5, 1, 1, 0.75, 0.5, 2, 0.9, 1.3, 0.8, 1.4, 1.1, 1),
        b1 = c(-2, -2, -2, -2.5, -2, -1.5, -1, -2.75, -1.8, -2.3, -2.6, -2.2, -2.2, -1.7, -1.4),
        b2 = c(-1, -1, -1, -2, -0.5, 0, 1, -1.5, -1.2, -1.7, -1.4, -1.1, -1.1, -0.6, -0.1),
        b3 = c(0, 0, 0, -1, 1, 1.5, 2, 1.5, 0.5, 2, 1.3, 1.4, 1.3, 0.8, 0.9),
        b4 = c(1.5, 1.5, 1.5, 1, 2, 2, 3, 2, 1.8, 2.5, 1.9, 2.5, 2.1, 1.6, 1.9)
      ),
      mod = "grm"
```

```
    )
  })
```

Create the questionnaire data.frame:

```
group_2_cat_1 <- group_2_irt_1 %>%
  map(function(data) {
    res <- as.data.frame(data$resp)
    colnames(res) <- paste0("q1_", 1:15)
    res
  })

group_2_questionnaire_1 <- map2(group_2_latent_1, group_2_cat_1,
                                function(latent, categorical) {
                                  bind_cols(
                                    latent %>%
                                      select(-c(y, y_norm)),
                                    categorical
                                  ) %>%
                                    mutate(across(starts_with("q"), as.factor))
                                })
```

1.2.1.2 Questionnaire 2

Create questionnaire 2 with 20 items and 4 levels each:

```
group_2_irt_2 <- group_2_latent_2 %>%
  map(function(data) {
    simIrt(
      theta = data$y_norm,
      params = params_q2,
      mod = "grm"
    )
  })
```

Create the questionnaire data.frame:

```
group_2_cat_2 <- group_2_irt_2 %>%
  map(function(data) {
    res <- as.data.frame(data$resp)
    colnames(res) <- paste0("q2_", 1:20)
    res
  })

group_2_questionnaire_2 <- map2(group_2_latent_2, group_2_cat_2,
                                function(latent, categorical) {
                                  bind_cols(
                                    latent %>%
                                      select(-c(y, y_norm)),
                                    categorical
                                  ) %>%
                                    mutate(across(starts_with("q"), as.factor))
                                })
```

1.2.1.3 Questionnaire 3

Create questionnaire 3 with 2 items and 5 levels each:

```r
group_2_irt_3 <- group_2_latent_3 %>%
  map(function(data) {
    simIrt(
      theta = data$y_norm,
      params = cbind(
        a = c(1, 1.5),
        b1 = c(-1.9, -1.3),
        b2 = c(-0.4, -0.1),
        b3 = c(0.9, 0.5),
        b4 = c(1.6, 1.8)
      ),
      mod = "grm"
    )
  })
```

Create the questionnaire data.frame and add the continuous variables:

```r
group_2_cat_3 <- group_2_irt_3 %>%
  map(function(data) {
    res <- as.data.frame(data$resp)
    colnames(res) <- paste0("q3_", 1:2)
    res
  })

group_2_questionnaire_3 <- pmap(list(group_2_latent_3, group_2_cat_3,
                                     group_2_continuous_3_1,
                                     group_2_continuous_3_2,
                                     group_2_continuous_3_3),
                                function(latent, categorical, cont_1,
                                         cont_2, cont_3) {
                                  bind_cols(
                                    latent %>%
                                      select(-c(y, y_norm)),
                                    categorical
                                  ) %>%
                                    mutate(across(starts_with("q"), as.factor)) %>%
                                    bind_cols(
                                      cont_1 %>% select(q3_3 = y_norm),
                                      cont_2 %>% select(q3_4 = y_norm),
                                      cont_3 %>% select(q3_5 = y_norm)
                                    )
                                })
```

1.2.2 3 groups

1.2.2.1 Questionnaire 1

Create questionnaire 1 with 15 items and 5 levels each:

```r
group_3_irt_1 <- group_3_latent_1 %>%
  map(function(data) {
    simIrt(
      theta = data$y_norm,
      params = cbind(
        a = c(0.5, 1, 1.5, 1.5, 1, 1, 0.75, 0.5, 2, 0.9, 1.3, 0.8, 1.4, 1.1, 1),
        b1 = c(-2, -2, -2, -2.5, -2, -1.5, -1, -2.75, -1.8, -2.3, -2.6, -2.2, -2.2, -1.7, -1.4),
        b2 = c(-1, -1, -1, -2, -0.5, 0, 1, -1.5, -1.2, -1.7, -1.4, -1.1, -1.1, -0.6, -0.1),
```

```
    b3 = c(0, 0, 0, -1, 1, 1.5, 2, 1.5, 0.5, 2, 1.3, 1.4, 1.3, 0.8, 0.9),
    b4 = c(1.5, 1.5, 1.5, 1, 2, 2, 3, 2, 1.8, 2.5, 1.9, 2.5, 2.1, 1.6, 1.9)
  ),
  mod = "grm"
 )
})
```

Create the questionnaire data.frame:

```
group_3_cat_1 <- group_3_irt_1 %>%
  map(function(data) {
    res <- as.data.frame(data$resp)
    colnames(res) <- paste0("q1_", 1:15)
    res
  })

group_3_questionnaire_1 <- map2(group_3_latent_1, group_3_cat_1,
                                function(latent, categorical) {
                                  bind_cols(
                                    latent %>%
                                      select(-c(y, y_norm)),
                                    categorical
                                  ) %>%
                                    mutate(across(starts_with("q"), as.factor))
                                })
```

1.2.2.2 Questionnaire 2

Create questionnaire 2 with 20 items and 4 levels each:

```
group_3_irt_2 <- group_3_latent_2 %>%
  map(function(data) {
    simIrt(
      theta = data$y_norm,
      params = params_q2,
      mod = "grm"
    )
  })
```

Create the questionnaire data.frame:

```
group_3_cat_2 <- group_3_irt_2 %>%
  map(function(data) {
    res <- as.data.frame(data$resp)
    colnames(res) <- paste0("q2_", 1:20)
    res
  })

group_3_questionnaire_2 <- map2(group_3_latent_2, group_3_cat_2,
                                function(latent, categorical) {
                                  bind_cols(
                                    latent %>%
                                      select(-c(y, y_norm)),
                                    categorical
                                  ) %>%
                                    mutate(across(starts_with("q"), as.factor))
                                })
```

1.2.2.3 Questionnaire 3

Create questionnaire 3 with 2 items and 5 levels each:

```
group_3_irt_3 <- group_3_latent_3 %>%
  map(function(data) {
    simIrt(
      theta = data$y_norm,
      params = cbind(
        a = c(1, 1.5),
        b1 = c(-1.9, -1.3),
        b2 = c(-0.4, -0.1),
        b3 = c(0.9, 0.5),
        b4 = c(1.6, 1.8)
      ),
      mod = "grm"
    )
  })
```

Create the questionnaire data.frame and add the continuous variables:

```
group_3_cat_3 <- group_3_irt_3 %>%
  map(function(data) {
    res <- as.data.frame(data$resp)
    colnames(res) <- paste0("q3_", 1:2)
    res
  })

group_3_questionnaire_3 <- pmap(list(group_3_latent_3, group_3_cat_3,
                                     group_3_continuous_3_1,
                                     group_3_continuous_3_2,
                                     group_3_continuous_3_3),
                                function(latent, categorical, cont_1,
                                         cont_2, cont_3) {
                                  bind_cols(
                                    latent %>%
                                      select(-c(y, y_norm)),
                                    categorical
                                  ) %>%
                                    mutate(across(starts_with("q"), as.factor)) %>%
                                    bind_cols(
                                      cont_1 %>% select(q3_3 = y_norm),
                                      cont_2 %>% select(q3_4 = y_norm),
                                      cont_3 %>% select(q3_5 = y_norm)
                                    )
                                })
```

1.2.3 4 groups

1.2.3.1 Questionnaire 1

Create questionnaire 1 with 15 items and 5 levels each:

```
group_4_irt_1 <- group_4_latent_1 %>%
  map(function(data) {
    simIrt(
      theta = data$y_norm,
      params = cbind(
        a = c(0.5, 1, 1.5, 1.5, 1, 1, 0.75, 0.5, 2, 0.9, 1.3, 0.8, 1.4, 1.1, 1),
        b1 = c(-2, -2, -2, -2.5, -2, -1.5, -1, -2.75, -1.8, -2.3, -2.6, -2.2, -2.2, -1.7, -1.4),
```

```
      b2 = c(-1, -1, -1, -2, -0.5, 0, 1, -1.5, -1.2, -1.7, -1.4, -1.1, -1.1, -0.6, -0.1),
      b3 = c(0, 0, 0, -1, 1, 1.5, 2, 1.5, 0.5, 2, 1.3, 1.4, 1.3, 0.8, 0.9),
      b4 = c(1.5, 1.5, 1.5, 1, 2, 2, 3, 2, 1.8, 2.5, 1.9, 2.5, 2.1, 1.6, 1.9)
    ),
    mod = "grm"
  )
})
```

Create the questionnaire data.frame:

```
group_4_cat_1 <- group_4_irt_1 %>%
  map(function(data) {
    res <- as.data.frame(data$resp)
    colnames(res) <- paste0("q1_", 1:15)
    res
  })

group_4_questionnaire_1 <- map2(group_4_latent_1, group_4_cat_1,
                                function(latent, categorical) {
                                  bind_cols(
                                    latent %>%
                                      select(-c(y, y_norm)),
                                    categorical
                                  ) %>%
                                    mutate(across(starts_with("q"), as.factor))
                                })
```

1.2.3.2 Questionnaire 2 Create questionnaire 2 with 20 items and 4 levels each:

```
group_4_irt_2 <- group_4_latent_2 %>%
  map(function(data) {
    simIrt(
      theta = data$y_norm,
      params = params_q2,
      mod = "grm"
    )
  })
```

Create the questionnaire data.frame:

```
group_4_cat_2 <- group_4_irt_2 %>%
  map(function(data) {
    res <- as.data.frame(data$resp)
    colnames(res) <- paste0("q2_", 1:20)
    res
  })

group_4_questionnaire_2 <- map2(group_4_latent_2, group_4_cat_2,
                                function(latent, categorical) {
                                  bind_cols(
                                    latent %>%
                                      select(-c(y, y_norm)),
                                    categorical
                                  ) %>%
                                    mutate(across(starts_with("q"), as.factor))
```

```
                                })
```

1.2.3.3 Questionnaire 3

Create questionnaire 3 with 2 items and 5 levels each:

```
group_4_irt_3 <- group_4_latent_3 %>%
  map(function(data) {
    simIrt(
      theta = data$y_norm,
      params = cbind(
        a = c(1, 1.5),
        b1 = c(-1.9, -1.3),
        b2 = c(-0.4, -0.1),
        b3 = c(0.9, 0.5),
        b4 = c(1.6, 1.8)
      ),
      mod = "grm"
    )
  })
```

Create the questionnaire data.frame and add the continuous variables:

```
group_4_cat_3 <- group_4_irt_3 %>%
  map(function(data) {
    res <- as.data.frame(data$resp)
    colnames(res) <- paste0("q3_", 1:2)
    res
  })

group_4_questionnaire_3 <- pmap(list(group_4_latent_3, group_4_cat_3,
                                     group_4_continuous_3_1,
                                     group_4_continuous_3_2,
                                     group_4_continuous_3_3),
                                function(latent, categorical, cont_1,
                                         cont_2, cont_3) {
                                  bind_cols(
                                    latent %>%
                                      select(-c(y, y_norm)),
                                    categorical
                                  ) %>%
                                    mutate(across(starts_with("q"), as.factor)) %>%
                                    bind_cols(
                                      cont_1 %>% select(q3_3 = y_norm),
                                      cont_2 %>% select(q3_4 = y_norm),
                                      cont_3 %>% select(q3_5 = y_norm)
                                    )
                                })
```

1.3 Create one data source

```
group_2_simulated_data <- pmap(list(group_2_questionnaire_1,
                                    group_2_questionnaire_2,
                                    group_2_questionnaire_3,
                                    group_2_continuous_cr),
                               function(q_1, q_2, q_3, crosssectional) {
```

```r
                                 bind_cols(
                                   q_1,
                                   q_2 %>% select(starts_with("q2")),
                                   q_3 %>% select(starts_with("q3"))
                                 ) %>%
                                   left_join(crosssectional %>%
                                               select(ID, additional_variable = y),
                                             by = "ID") %>%
                                   left_join(age_df, by = "ID")
                               })

group_3_simulated_data <- pmap(list(group_3_questionnaire_1,
                                    group_3_questionnaire_2,
                                    group_3_questionnaire_3,
                                    group_3_continuous_cr),
                               function(q_1, q_2, q_3, crosssectional) {
                                 bind_cols(
                                   q_1,
                                   q_2 %>% select(starts_with("q2")),
                                   q_3 %>% select(starts_with("q3"))
                                 ) %>%
                                   left_join(crosssectional %>%
                                               select(ID, additional_variable = y),
                                             by = "ID") %>%
                                   left_join(age_df, by = "ID")
                               })

group_4_simulated_data <- pmap(list(group_4_questionnaire_1,
                                    group_4_questionnaire_2,
                                    group_4_questionnaire_3,
                                    group_4_continuous_cr),
                               function(q_1, q_2, q_3, crosssectional) {
                                 bind_cols(
                                   q_1,
                                   q_2 %>% select(starts_with("q2")),
                                   q_3 %>% select(starts_with("q3"))
                                 ) %>%
                                   left_join(crosssectional %>%
                                               select(ID, additional_variable = y),
                                             by = "ID") %>%
                                   left_join(age_df, by = "ID")
                               })
```

```r
saveRDS(group_2_simulated_data,
        "03_data/simulated_dataset_different_n_group_2_2024_01_29.rds")
saveRDS(group_3_simulated_data,
        "03_data/simulated_dataset_different_n_group_3_2024_01_29.rds")
saveRDS(group_4_simulated_data,
        "03_data/simulated_dataset_different_n_group_4_2024_01_29.rds")
```

2 Run the simulation

The following is the code executed on the command line with the arguments for the different number of groups and n:

```r
args = commandArgs(trailingOnly=TRUE)
number_groups <- as.character(args[1])
number_obs <- as.character(args[2])

library(dplyr)
library(FactoMineR)
library(factoextra)
library(longmixr)
library(flexmix)
library(lme4)
library(purrr)

# process the data
data <- readRDS(paste0("simulated_dataset_different_n_group_", number_groups, "_2024_01_29.rds"))

# Dimension reduction
# do the dimension reduction by group
# groups:
# - questionnaire_1
# - questionnaire_2
# - questionnaire_3

q1_dim <- data %>%
  set_names(nm = c(50, 100, 200, 500, 1000)) %>%
  map(function(data) {
    data %>%
      select(starts_with("q1")) %>%
      FAMD(ncp = 10, graph = FALSE)
  })

q2_dim <- data %>%
  set_names(nm = c(50, 100, 200, 500, 1000)) %>%
  map(function(data) {
    data %>%
      select(starts_with("q2")) %>%
      FAMD(ncp = 10, graph = FALSE)
  })

q3_dim <- data %>%
  set_names(nm = c(50, 100, 200, 500, 1000)) %>%
  map(function(data) {
    data %>%
      select(starts_with("q3")) %>%
      FAMD(ncp = 10, graph = FALSE)
  })

# Data wrangling
# Bring the data into a format suitable for flexmix
```

```r
# look how much variance the components explain and use the elbow plot method
# to decide on the number of components -> 2 for all settings

# use components 1-2
q1_comp <- as.data.frame(q1_dim[[number_obs]]$ind$coord[, 1:2])
colnames(q1_comp) <- paste0("q1_", 1:2)
# use components 1-2
q2_comp <- as.data.frame(q2_dim[[number_obs]]$ind$coord[, 1:2])
colnames(q2_comp) <- paste0("q2_", 1:2)
# use components 1-2
q3_comp <- as.data.frame(q3_dim[[number_obs]]$ind$coord[, 1:2])
colnames(q3_comp) <- paste0("q3_", 1:2)

cluster_data <- cbind(data.frame(
  patient_id = data[[number_obs]]$ID,
  visit = data[[number_obs]]$visit
),
q1_comp,
q2_comp,
q3_comp
)

# add the age information
age_info <- data[[number_obs]] %>%
  select(ID, age = age_visit_1) %>%
  distinct(ID, .keep_all = TRUE)

# regress out the age information
generate_residuals <- function(x, age, patient_id) {
  data <- data.frame(
    x = x,
    age = age,
    patient_id = patient_id)
  model <- lmer(x ~ age + (1 | patient_id), data = data[[number_obs]])
  resid <- residuals(model, type = "response")
  names(resid) <- NULL
  resid
}

cluster_data_age <- cluster_data %>%
  left_join(age_info, by = c("patient_id" = "ID"))

cluster_data_resid <- cluster_data_age %>%
  mutate(across(matches("[1-9]$"),
                ~generate_residuals(x = .x, age = age, patient_id = patient_id),
                .names = "{.col}_resid")) %>%
  select(patient_id, visit, ends_with("resid"), age)


# set up the separate terms for the responses
response_names <- c(paste0("q1_", 1:2, "_resid"),
                    paste0("q2_", 1:2, "_resid"),
                    paste0("q3_", 1:2, "_resid"))
```

```r
list_models <- lapply(response_names, function(x) {
  FLXMRmgcv(as.formula(paste0(x, " ~ .")))
})

model <- longitudinal_consensus_cluster(data = cluster_data_resid,
                                        id_column = "patient_id",
                                        max_k = 5,
                                        reps = 100,
                                        p_item = 0.8,
                                        model_list = list_models,
                                        flexmix_formula = as.formula("~s(visit, k = 4) | patient_id"),
                                        final_linkage = "ward.D2")

saveRDS(model, paste0("lcc_model_simulated_data_group_",
                      number_groups, "_n_", number_obs, "_2024_01_29.Rds"))
```
