# Supplementary material for: longmixr: a tool for robust clustering of high-dimensional cross-sectional and longitudinal variables of mixed data types
Source: Bioinformatics. 2024 Mar 14;40(4):btae137. doi: 10.1093/bioinformatics/btae137 (PMC10994717; doi:10.1093/bioinformatics/btae137)
Supplement: btae137_Supplementary_Data [file btae137_supplementary_data.zip › supplementary_longmixr_Hagenberg.pdf]

Supplementary for ‘longmixr: A tool for robust clustering of high-dimensional cross-sectional and longitudinal phenotype data’

Jonas Hagenberg Monika Budde Teodora Pandeva Ivan Kondofersky
Sabrina K. Schaupp Fabian J. Theis Thomas G. Schulze Nikola S. Müller
Urs Heilbronner Richa Batra Janine Knauer-Arloth

Contents

1 Overview 1

2 Methods 1
2.1 Consensus clustering for longitudinal data . . . 1
2.2 Consensus clustering for cross-sectional categorical data . . . 2

3 Supplemental data 3
3.1 Application of longmixr on simulated data . . . 3
3.2 Application of longmixr on real data . . . 12

References 32

1 Overview

The supplementary is structured as follows: First, an explanation about the clustering for cross-sectional data is given. Afterwards, additional details for the case study with longitudinal data from the PsyCourse Study are given.

2 Methods

2.1 Consensus clustering for longitudinal data

Longmixr uses flexmix which is based on finite mixture models (Grün and Leisch, 2020). In short, this assumes that the observed data results from k distributions or components with D independent variables as a multivariate outcome where each distribution is present with a certain fraction:

$$h(y|x, \psi) = \sum_{k=1}^{K} \pi_k f(y|x, \theta_k) = \sum_{k=1}^{K} \pi_k \prod_{d=1}^{D} f_d(y|x, \theta_{kd}) \pi_k \geq 0, \sum_{k=1}^{K} \pi_k = 1.$$

For longmixr, $y = (y_1, ..., y_D)^T$ are the components from the dimensionality reduced mixed data that has the conditional density h and x is a vector of independent variables, in this case the time variable that is modeled as a smooth effect with splines. The prior probability of component k is given by π_k, θ_{kd} contains the parameters specific for the component with density function f_{kd} and ψ is the vector of all parameters.

As longmixr deals with N_m repeated measurements from an individual m, having M individuals the log-likelihood is

$$\log L = \sum_{m=1}^{M}\sum_{n=1}^{N_m} \log h(y_{mn}|x_{mn},\psi) = \sum_{m=1}^{M}\sum_{n=1}^{N_m} \log(\sum_{k=1}^{K} \pi_k f(y_{mn}|x_{mn},\theta_k)), \sum_{m=1}^{M} N_m = N.$$

Then, the posterior probability that an individual m with (x_{mn}, y_{mn}) as the nth observation belongs to component or cluster j is

$$P(j|m) = \frac{\pi_j \prod_{n=1}^{N_m} f(y_{mn}|x_{mn},\theta_j)}{\sum_k \pi_k \prod_{n=1}^{N_m} f(y_{mn}|x_{mn},\theta_k)}.$$

This posterior probability is used to assign cluster labels to the observations. The parameters are estimated with the expectation-maximization (EM) algorithm, for a detailed overview see Leisch (2004).

For the consensus clustering, the above modeling is repeated G times on a subset of the data, $D^{(g)}$. The full data set has N individuals, while $D^{(g)}$ has fewer individuals. Let $M^{(g)}$ be the $N \times N$ connectivity matrix for data set $D^{(g)}$ defined as

$$M^{(g)}(i,j) = \begin{cases} 1 & \text{if individuals } i \text{ and } j \text{ belong to the same cluster,} \\ 0 & \text{otherwise} \end{cases}$$

and the $N \times N$ indicator matrix $I^{(g)}$ as

$$I^{(g)}(i,j) = \begin{cases} 1 & \text{if individuals } i \text{ and } j \text{ are present in the data set } D^{(g)}, \\ 0 & \text{otherwise} \end{cases}.$$

In the following, the indicator matrix can be used to correctly normalise the consensus matrix as not all individuals are contained in all subsets of the data.

Finally, the consensus matrix can be calculated as

$$C(i,j) = \frac{\sum_g M^{(g)}(i,j)}{\sum_g I^{(g)}(i,j)}.$$

The consensus matrix contains the information how often two individuals are clustered into the same cluster across the subsets. For a detailed overview, see Monti *et al.* (2003).

2.2 Consensus clustering for cross-sectional categorical data

2.2.1 Gower distance

The Gower distance is a measure to compute dissimilarity between entities based on mixed data types (equations 1-3).

$$d_{ij} = \frac{\sum_k \delta_{ijk} d_{ijk}}{\sum_k \delta_{ijk}} \tag{1}$$

$$d_{ijk} = \frac{|x_{ik} - x_{jk}|}{R_k} \tag{2}$$

$$z_{ik} = \frac{(r_{ik} - 1)}{\max(r_{ik}) - 1} \tag{3}$$

D_{ij} in equation 1 represents the distance between sample i and j across all variables. d_{ijk} is the distance for the specific variable k and is dependent on the nature or type of the variable and δ_{ijk} an additional weight. d_{ijk} has the following rules:

- for asymmetric binary variables, if $x_{ik} = x_{jk}$, d_{ijk} is 0, else 1
- for categorical nominal variables, if $x_{ik} = x_{jk}$, d_{ijk} is 0, else 1
- numeric variables are considered as interval-scaled variables and the distance for one variable k is calculated according to equation 2. R_k is the range of the variable k
- for categorical ordinal variables, the values are replaced by their corresponding ranks in the factor levels according to equation 3 where r_{ik} is the rank.

The additional weight δ_{ijk} follows the following rules:

- δ_{ijk} is 0 if x_{ik} or x_{jk} is NA
- δ_{ijk} is 0 if k is an asymmetric binary variable and both x_{ik} and x_{jk} are 0 (or FALSE)
- in all other cases, δ_{ijk} is 1

There are several packages in R to compute gower distance, e.g. FD (Laliberté and Legendre, 2010; Laliberté *et al.*, 2014), cluster (Maechler *et al.*, 2021) and StatMatch (D'Orazio, 2022).

2.2.2 Consensus clustering for cross-sectional data

The package ConsensusClusterPlus implements consensus clustering in R (Wilkerson and Hayes, 2010, 2020) with hierarchical clustering. We developed a workflow to use the package with cross-sectional data that includes categorical features. First, the data is converted into a distance measure using the Gower distance. The Gower distance is a measure to compute dissimilarity between entities based on mixed data types. We used the gower.dist function from the R package StatMatch (D'Orazio, 2022). For the linkage function we chose ward.D2 as it is a commonly used linkage function that can also be applied to non-Euclidean distances (Miyamoto *et al.*, 2015). As hierarchical clustering is not very computationally intensive, we use 1000 repetitions and apply the ConsensusClusterPlus default subsampling rate of 0.8. This complete workflow is implemented in the wrapper function `crosssectional_consensus_cluster` that calls ConsensusClusterPlus with the above mentioned settings.

3 Supplemental data

3.1 Application of longmixr on simulated data

3.1.1 Data simulation

The data is simulated in two steps:

1. generate a continuous variable from a mixed model
2. use this variable as theta (latent variable or ability) in a graded response model (GRM)

The theta in the GRM should follow a N(0, 1) distribution. Therefore, the values from step 1 are mapped to the quantiles of a N(0, 1) function. For every questionnaire, one mixed model is used. In the GRM, the number of the items per questionnaire, the discrimination of each item and the difficulty/ threshold for every class (the value of theta at which the probability to select the next class is 50%) can be varied.

In total, 50, 100, 200, 500 and 1000 individuals aged between 18 - 65 with 4 time points in 4 equally sized groups are simulated with 3 questionnaires:

- 15 items with 5 levels each
- 20 items with 4 levels each
- 2 items with 5 levels each and 3 continuous variables

Additionally, a cross-sectional continuous variable is simulated that could be used to compare the groups after clustering.

For the simulation code, see the supplementary_simulation_code_longmixr_Hagenberg.pdf file.

Load the required packages:

```r
library(longmixr)
library(FactoMineR)
library(factoextra)
library(flexmix)
library(purrr)
library(tidyr)
library(ggplot2)
library(ggalluvial)
library(lme4)
library(dplyr)
options(dplyr.summarise.inform = FALSE)
library(fossil, include.only = c("rand.index", "adj.rand.index"))
library(nlme)
library(kableExtra)
```

3.1.2 Results

Read in the results from the clustering on the simulated data:

```r
data_group_2 <- readRDS("03_data/simulated_dataset_different_n_group_2_2024_01_29.rds")
data_group_3 <- readRDS("03_data/simulated_dataset_different_n_group_3_2024_01_29.rds")
data_group_4 <- readRDS("03_data/simulated_dataset_different_n_group_4_2024_01_29.rds")

res_2 <- c(50, 100, 200, 500, 1000) %>%
  set_names() %>%
  map(~readRDS(paste0("03_data/01_results_from_cluster/lcc_model_simulated_data_group_2_n_",
                      .x, "_2024_01_29.Rds")))
res_3 <- c(50, 100, 200, 500, 1000) %>%
  set_names() %>%
  map(~readRDS(paste0("03_data/01_results_from_cluster/lcc_model_simulated_data_group_3_n_",
                      .x, "_2024_01_29.Rds")))
res_4 <- c(50, 100, 200, 500, 1000) %>%
  set_names() %>%
  map(~readRDS(paste0("03_data/01_results_from_cluster/lcc_model_simulated_data_group_4_n_",
                      .x, "_2024_01_29.Rds")))
```

The overlap between the ground truth groups and the results from the clustering is quantified by the rand index and shown in Table S1. For all n and group sizes, the rand index is the highest for the clustering with the correct number of clusters.

```r
data_simulation <- list(`2` = data_group_2, `3` = data_group_3, `4` = data_group_4)
results_simulation <- list(`2` = res_2, `3` = res_3, `4` = res_4)

calculate_rand <- function(original_data, results) {
  map2_dfr(original_data, results, function(original_data, results) {
  groundtruth <- get_clusters(results)
  res <- groundtruth %>%
    left_join(original_data %>% select(patient_id = ID, group) %>%
              distinct(patient_id, .keep_all = TRUE) %>%
              mutate(patient_id = as.factor(patient_id)), by = "patient_id")

  tibble::tibble(
    `rand index` = c(
      rand.index(as.numeric(res$group), res$assignment_num_clus_2),
      rand.index(as.numeric(res$group), res$assignment_num_clus_3),
```

```
      rand.index(as.numeric(res$group), res$assignment_num_clus_4),
      rand.index(as.numeric(res$group), res$assignment_num_clus_5)
    ),
    `clustering groups` = 2:5
  )
}, .id = "n")
}

map2_dfr(data_simulation, results_simulation, calculate_rand,
                       .id = "simulated groups") %>%
  kbl(
    booktabs = TRUE,
    caption = "Rand index of clustering results with ground truth groups for the simulated data.",
    longtable = TRUE
  )
```

Table S1: Rand index of clustering results with ground truth groups for the simulated data.

simulated groups	n	rand index	clustering groups
2	50	1.0000000	2
2	50	0.8726531	3
2	50	0.7616327	4
2	50	0.7093878	5
2	100	1.0000000	2
2	100	0.9321212	3
2	100	0.8529293	4
2	100	0.7430303	5
2	200	1.0000000	2
2	200	0.8804523	3
2	200	0.8000503	4
2	200	0.7268844	5
2	500	1.0000000	2
2	500	0.8747816	3
2	500	0.7495391	4
2	500	0.7401202	5
2	1000	1.0000000	2
2	1000	0.8921902	3
2	1000	0.7851291	4
2	1000	0.7259520	5
3	50	0.7648980	2
3	50	0.9730612	3
3	50	0.9469388	4
3	50	0.9273469	5
3	100	0.7337374	2
3	100	0.9474747	3
3	100	0.8961616	4
3	100	0.8820202	5
3	200	0.7646734	2
3	200	0.9673869	3
3	200	0.9139698	4

3	200	0.8813568	5
3	500	0.7764489	2
3	500	0.9765531	3
3	500	0.9332665	4
3	500	0.8817395	5
3	1000	0.7753333	2
3	1000	0.9740280	3
3	1000	0.9326046	4
3	1000	0.8808248	5
4	50	0.6081633	2
4	50	0.8628571	3
4	50	0.9436735	4
4	50	0.9151020	5
4	100	0.6795960	2
4	100	0.8737374	3
4	100	0.9703030	4
4	100	0.9608081	5
4	200	0.7075879	2
4	200	0.8377387	3
4	200	0.9851759	4
4	200	0.9641709	5
4	500	0.7358637	2
4	500	0.8698116	3
4	500	0.9786373	4
4	500	0.9465251	5
4	1000	0.7322122	2
4	1000	0.8540160	3
4	1000	0.9728148	4
4	1000	0.9485465	5

Besides the overlap with the ground truth, another important point is the selection of the appropriate number of clusters based on the clustering results. Longmixr offers several diagnostic plots. For simulated data with two clusters, the consensus CDF hints at the correct number already for n = 50 (Figure S1).

```
plot(res_2[["50"]], which_plots = "CDF")
```

For four clusters, the decision is more difficult. While the consensus CDF plot (Figure S2) could also suggest five clusters even for n = 1000, the consensus matrix shows that one cluster is quite small and the overall structure hints at four clusters (Figure S3). The consensus matrices for three and four clusters and the item consensus plot (Figures S4 - S6) are ambiguous, showing that the cluster selection for higher number of clusters becomes more difficult and that it is important to consult several diagnostic plots.

```
plot(res_4[["1000"]], which_plots = "CDF")
```

```
plot(res_4[["1000"]], which_plots = "consensusmatrix_5")
```

```
plot(res_4[["1000"]], which_plots = "consensusmatrix_3")
```

```
plot(res_4[["1000"]], which_plots = "consensusmatrix_4")
```

```
plot(res_4[["1000"]], which_plots = "item_consensus", n_item_consensus = 4)
```

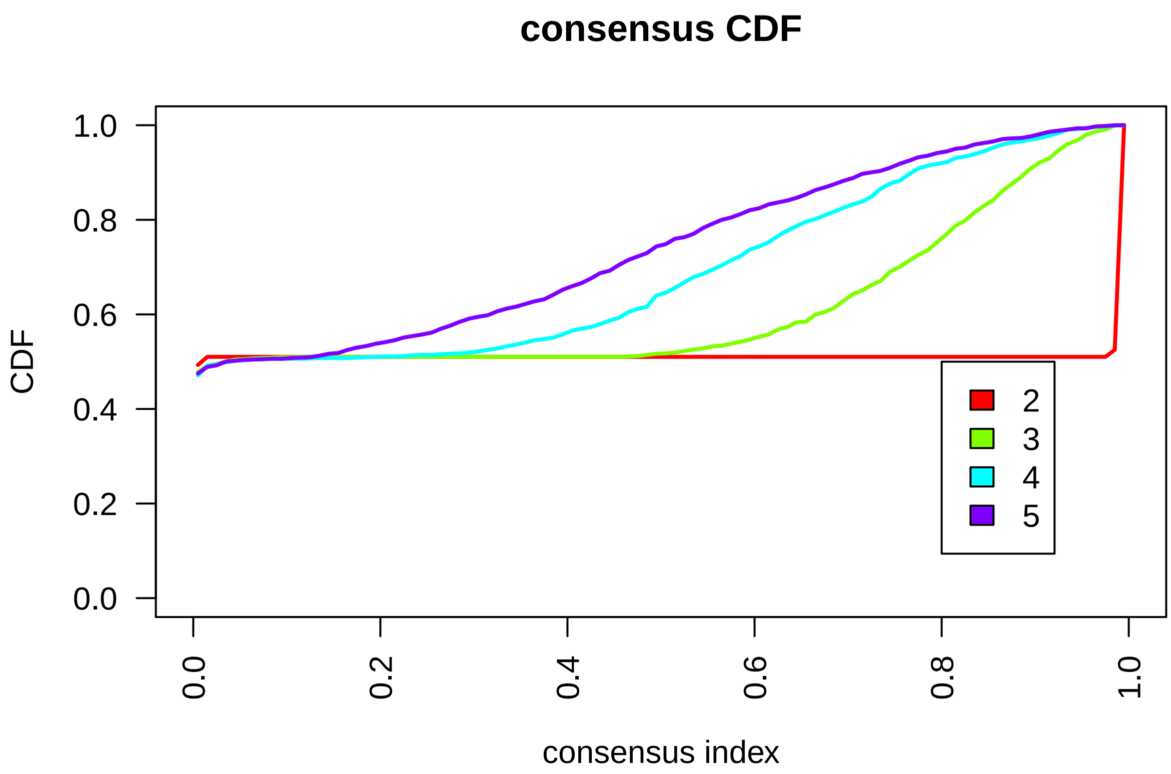

Figure S1: Consensus CDF for n = 50 simulated individuals with two groups.

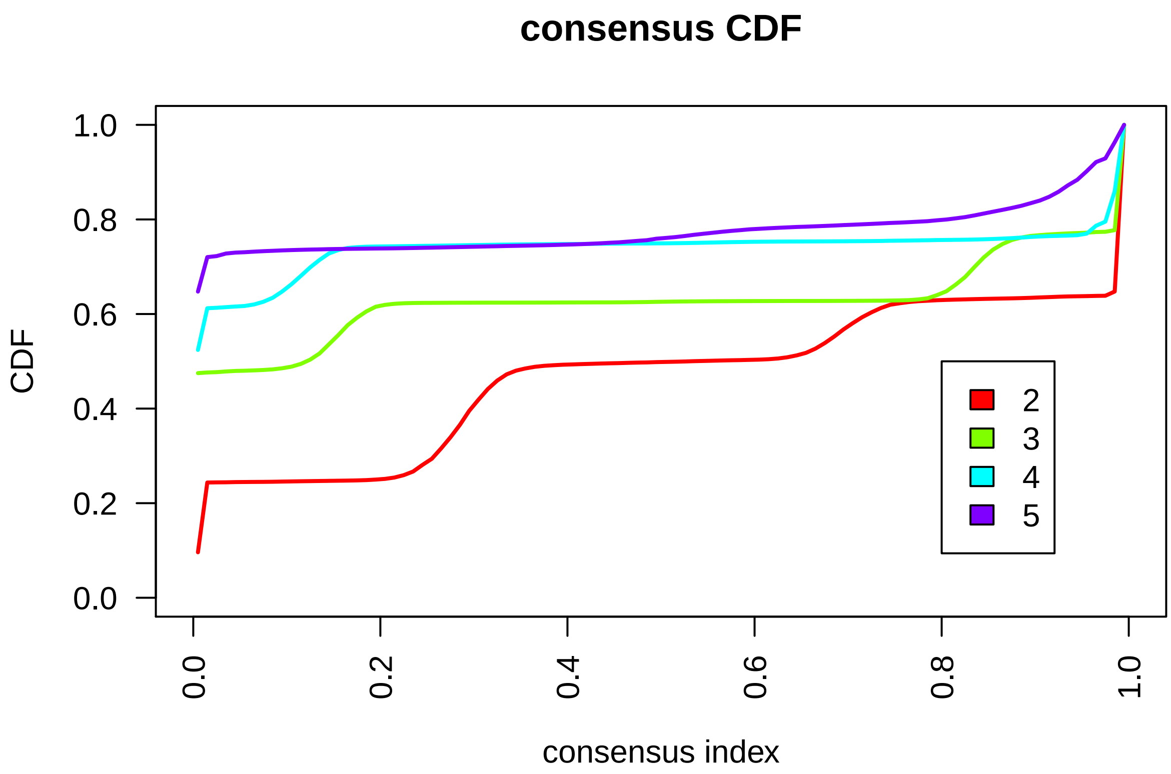

Figure S2: Consensus CDF for n = 1000 simulated individuals with four groups.

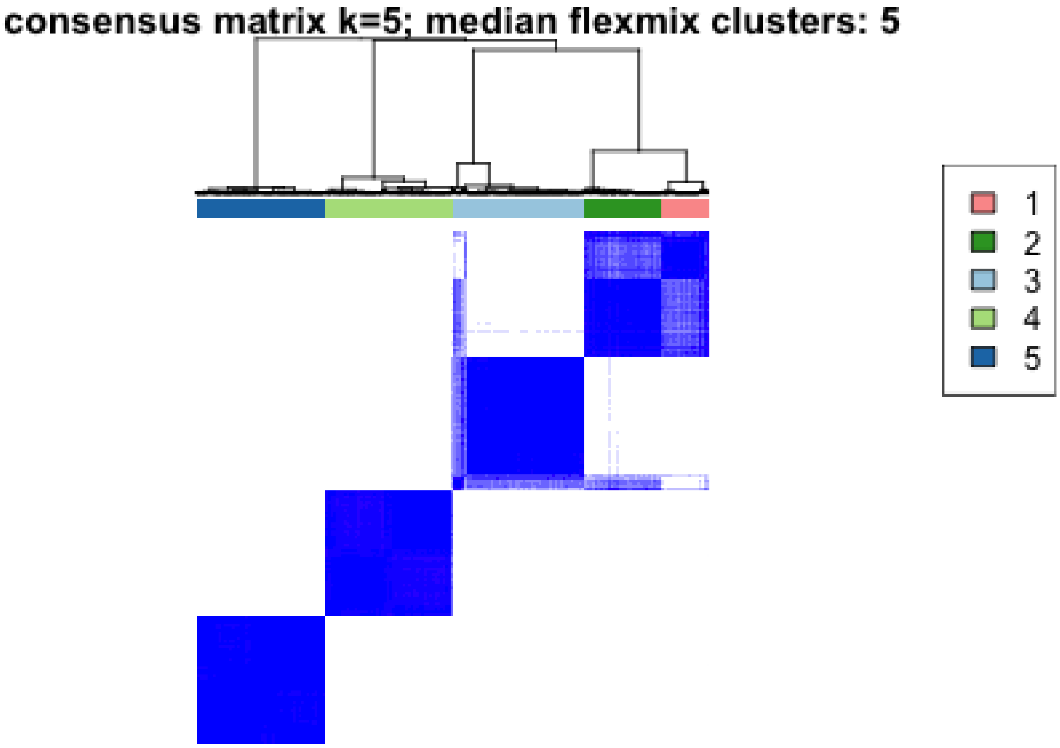

Figure S3: Consensus matrix for five clusters for n = 1000 simulated individuals with four groups.

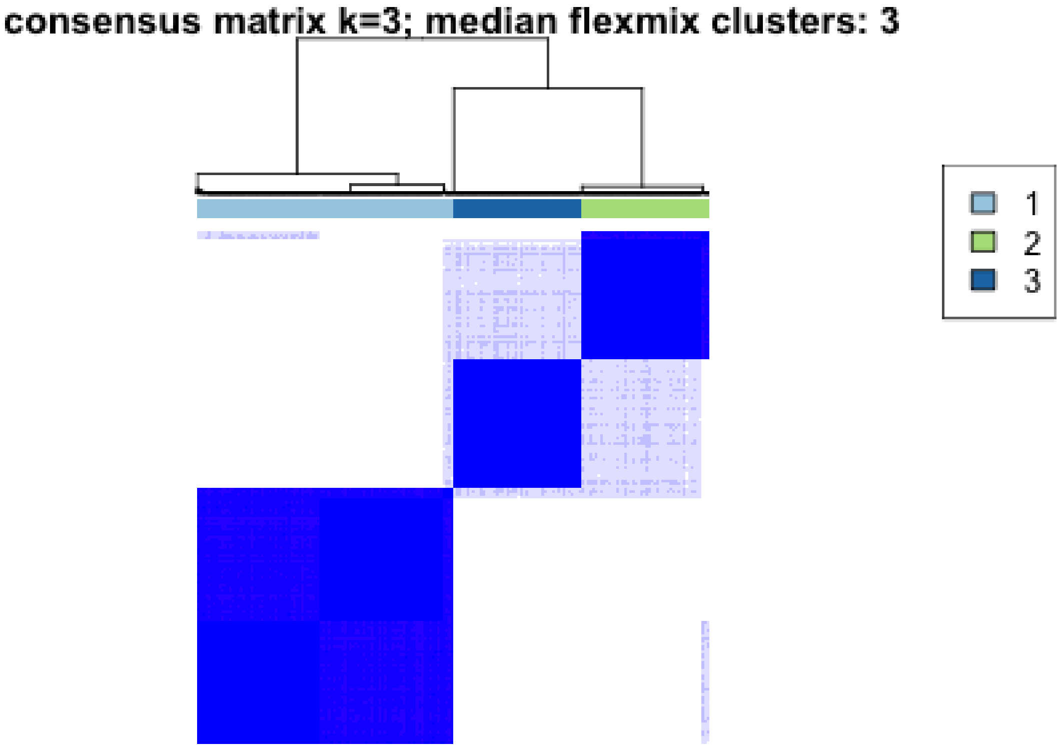

Figure S4: Consensus matrix for three clusters for n = 1000 simulated individuals with four groups.

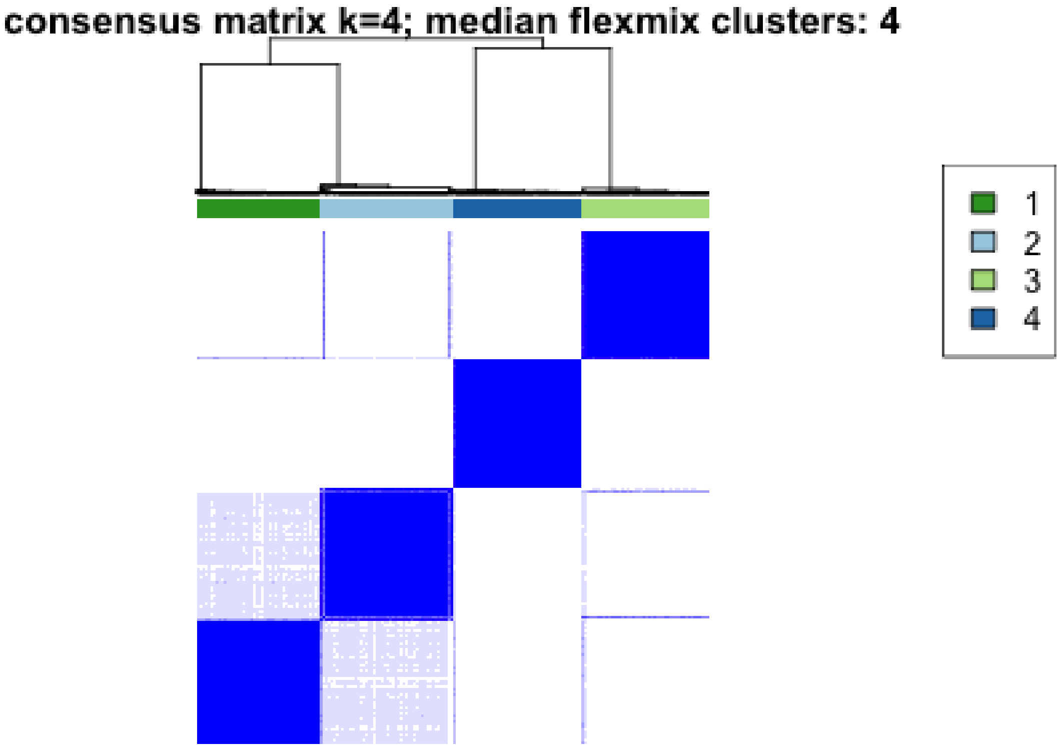

Figure S5: Consensus matrix for four clusters for n = 1000 simulated individuals with four groups.

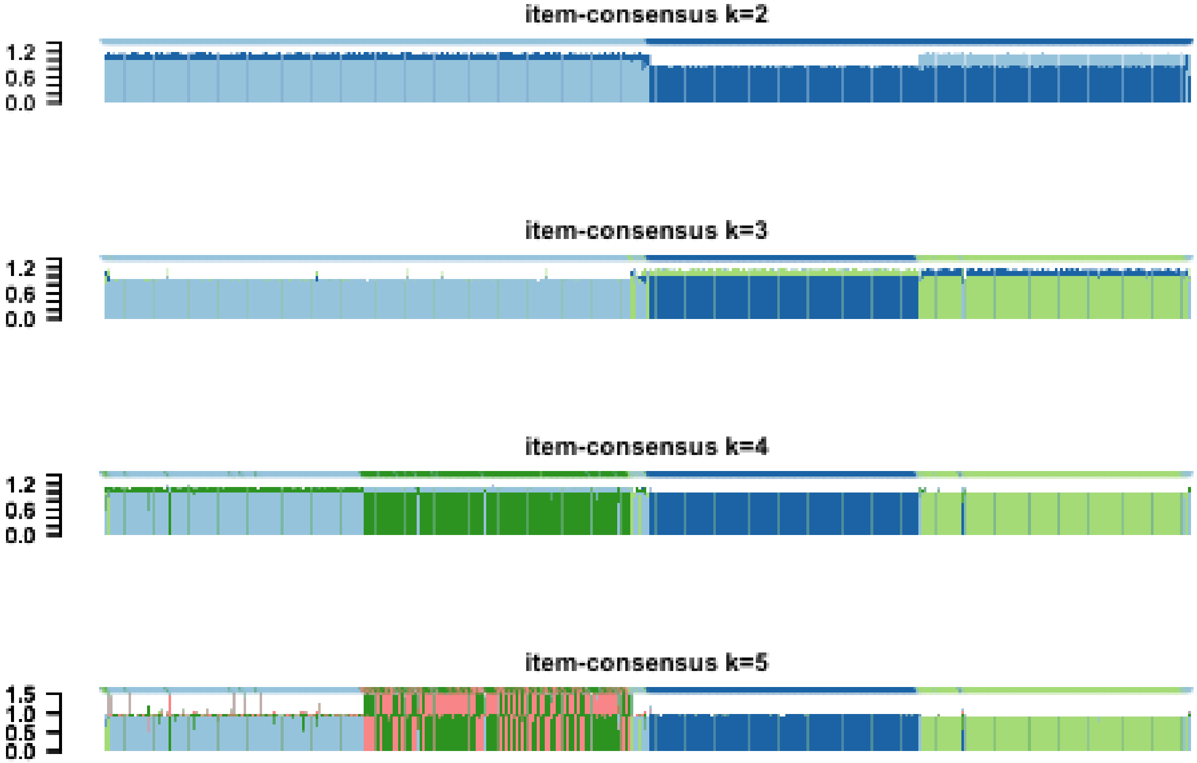

Figure S6: Item consensus for n = 1000 simulated individuals with four groups.

3.2 Application of longmixr on real data

The data used to illustrate the longitudinal part of the toolbox are from the PsyCourse Study (PMID: 30070057, Budde *et al.* (2019)), a transdiagnostic psychiatric study including clinical participants from the affective-to-psychotic spectrum as well as controls. For our purposes, we selected a subsample of study participants with a DSM-IV diagnosis of schizophrenia (SZ) and complete data at all four study visits (n=76). SZ is a heterogeneous illness regarding occurring symptoms and level of functioning (Kahn *et al.*, 2015). Many, but not all patients with SZ not only experience episodes of positive symptoms, such as delusions and hallucinations, but also suffer from negative and/or depressive symptoms. The latter are especially hard to treat and often have a high impact on the quality of life and level of functioning of patients. Therefore, we examined which clusters of individuals emerge from the PsyCourse data with regards to level of functioning and negative as well as depressive symptoms. Data have been collected at four equally distanced time points over a course of 18 months in total.

3.2.1 Data processing

We included variables from the Inventory of Depressive Symptoms (clinician rated, IDS-C_{30}, 30 items), from the Beck Depression Inventory (self-report, BDI-II, 21 items), items on negative symptoms of the Positive and Negative Syndrome Scale (clinician rated, PANSSneg, 7 items), demographic information about the current employment and relationship status (one item each), and the Global Assessment of Functioning score (clinician rated, GAF, one item). The latter assessment measures an individual's psychosocial functioning. Factor analysis of Mixed Data was used to reduce the dimension of the variables, grouped according to the different assessments:

1. Rater-Assessed Depressive Symptoms (IDS-C_{30})
2. Rater-Assessed Negative Symptoms (PANSSneg)
3. Self-Reported Depressive Symptoms (BDI-II)
4. Rater-Assessed Current Psychosocial Functioning (Relationship status, employment, GAF)

Missing values in longitudinal data were imputed using the R package Amelia (Honaker *et al.*, 2011, 2018) beforehand, and reverse items were adequately re-coded. If applicable, items were recoded so that higher values correspond to lower symptom burden and/or better functioning for better interpretability.

```
data <- read.csv("03_data/190917_dataset4publication.csv")
load("03_data/190225_v3.0_psycourse_long_format4clustering.RData")

# code variables as factors
data <- data %>%
  mutate(across(-c(gaf, v1_id, visit), as.factor),
         gaf = scale(gaf)) # scale the gaf variable
```

3.2.2 Dimension reduction

As we create a model for each variable, we need to reduce the dimensionality of the data set. For this, `FAMD` from the package FactoMineR (Lê *et al.*, 2008; Husson *et al.*, 2020) is used. This stands for Factor analysis of mixed data and is principal component method that can deal with both continuous and categorical variables.

The dimension reduction is performed in groups of variables belonging together. In this way the interpretation is easier. The groups are:

- idsc
- panss
- bdi
- the other three variables `curr_paid_empl`, `partner` and `gaf` (together also referred to as psychosocial functioning)

```
idsc_dim <- data %>%
  select(starts_with("idsc")) %>%
```

```r
  FAMD(ncp = 10, graph = FALSE)

panss_dim <- data %>%
  select(starts_with("panss")) %>%
  FAMD(ncp = 10, graph = FALSE)

bdi_dim <- data %>%
  select(starts_with("bdi")) %>%
  FAMD(ncp = 10, graph = FALSE)

other_dim <- data %>%
  select(curr_paid_empl, partner, gaf) %>%
  FAMD(ncp = 10, graph = FALSE)
```

The following components are chosen: The first three factors of the IDS-C_{30}, the first five factors of the PANSS negative scale, the first three factors of the BDI-II and the first two factors of the Psychosocial Functioning dimension, explaining 16.16%, 34.63%, 27.34% and 73.04% of the respective total variances of the dimensions. Therefore, we reduced the variable space from 61 single variables to 13 dimensions.

```r
idsc_comp <- as.data.frame(idsc_dim$ind$coord[, 1:3])
colnames(idsc_comp) <- paste0("idsc_", 1:3)
panss_comp <- as.data.frame(panss_dim$ind$coord[, 1:5])
colnames(panss_comp) <- paste0("panss_", 1:5)
bdi_comp <- as.data.frame(bdi_dim$ind$coord[, 1:3])
colnames(bdi_comp) <- paste0("bdi_", 1:3)
other_comp <- as.data.frame(other_dim$ind$coord[, 1:2])
colnames(other_comp) <- paste0("other_", 1:2)
cluster_data <- cbind(data.frame(
  patient_id = data$v1_id,
  visit = data$visit
),
idsc_comp,
panss_comp,
bdi_comp,
other_comp
)
```

3.2.3 Adjustment for covariates

Often, controlling for covariates in your data is important. For the longitudinal clustering, this can be achieved by regressing out the effect of the covariates from the components generated by the dimension reduction algorithm. Then, the residuals are used for the clustering. In case of the clustering of cross-sectional data, the user has to directly adjust for the covariates on the data level.

In the PsyCourse data, it is known that the age can have an influence on the measured variables. Therefore, the age is regressed out with a linear mixed model and for the following analysis, only the residuals are used.

```r
# add the age information
age_info <- psycrs3.0_lng %>%
  select(v1_id, age) %>%
  distinct(v1_id, .keep_all = TRUE)

# regress out the age information
generate_residuals <- function(x, age, patient_id) {
  data <- data.frame(
```

```
    x = x,
    age = age,
    patient_id = patient_id)
  model <- lmer(x ~ age + (1 | patient_id), data = data)
  resid <- residuals(model, type = "response")
  names(resid) <- NULL
  resid
}

cluster_data_age <- cluster_data %>%
  left_join(age_info, by = c("patient_id" = "v1_id"))

cluster_data_resid <- cluster_data_age %>%
  mutate(across(matches("[1-9]$"),
                ~generate_residuals(x = .x, age = age, patient_id = patient_id),
                .names = "{.col}_resid")) %>%
  select(patient_id, visit, ends_with("resid"), age)
```

Data after the age effect is regressed out:

```
cluster_data_resid_long <- cluster_data_resid %>%
  pivot_longer(
    -c(patient_id, visit, age),
    names_to = "variable",
    values_to = "residuals"
  )

ggplot(cluster_data_resid_long, aes(x = age, y = residuals)) +
  geom_point() +
  facet_wrap(~variable) +
  theme_bw()
```

3.2.4 Longitudinal consensus clustering

For the clustering, the modelling strategy is as follows. Every principal component from the data set with reduced dimensions is used in a flexible mixture model as an outcome variable, which can be seen as a multivariate outcome. This outcome only depends on the time, which is included with a spline basis which allows for a smooth modelling. Additionally, the model controls for the repeated measurements resulting from the different time points.

The mixture modelling tries to find several distributions from which the observed data points come from. The assigned distributions are the different clusters.

For robustness, the clustering is repeated n times on a subset of the data and for different numbers of clusters. Then it is counted how often each pair of observations are assigned the same cluster (for every time point the same cluster is assigned, therefore one only looks at pairs of subjects). On this "consensus matrix" one can define a criterion to find the optimal number of clusters. On this consensus cluster one carries out a hierarchical clustering step to assign the final clusters.

3.2.4.1 Define the flexmix models For the flexible mixture models, we use the package flexmix (Grün and Leisch, 2008, 2020).

```
# set up the separate terms for the responses
response_names <- c(paste0("idsc_", 1:3, "_resid"),
                    paste0("panss_", 1:4, "_resid"),
                    paste0("bdi_", 1:2, "_resid"),
```

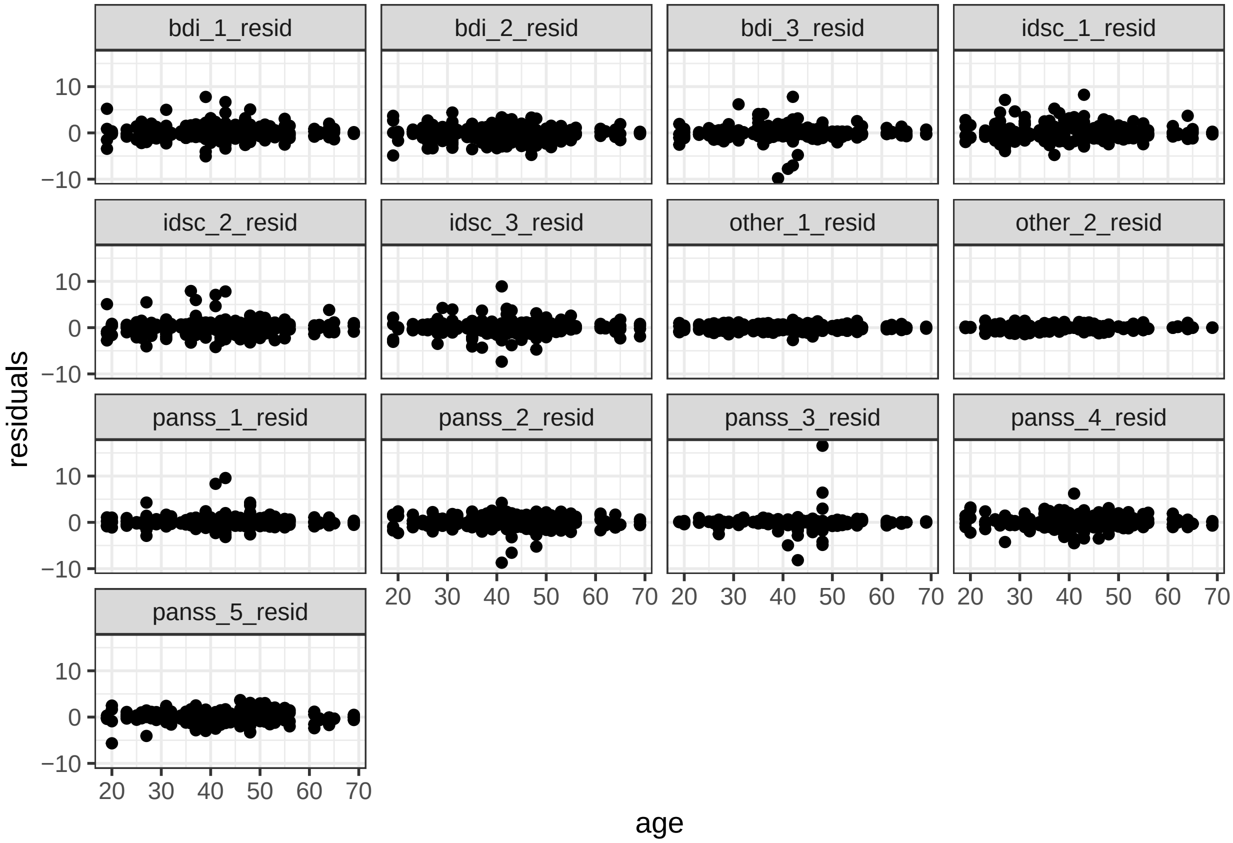

Figure S7: Residuals of the components after regressing out the age.

```
                     paste0("other_", 1, "_resid"))

list_models <- lapply(response_names, function(x) {
  FLXMRmgcv(as.formula(paste0(x, " ~ .")))
})
```

3.2.4.2 Consensus clustering The clustering takes some hours to compute, therefore it was done on a cluster and only the results loaded.

```
# model <- longitudinal_consensus_cluster(data = cluster_data_resid,
#                                          id_column = "patient_id",
#                                          max_k = 6,
#                                          reps = 100,
#                                          p_item = 0.8,
#                                          model_list = list_models,
#                                          flexmix_formula =
#                                            as.formula("~s(visit, k = 4) | patient_id"),
#                                          final_linkage = "ward.D2")

model <- readRDS("03_data/01_results_from_cluster/lcc_model_2024_02_01.Rds")
```

3.2.5 Results

3.2.5.1 Analysis plots to determine number of clusters To determine the optimal number of clusters, longmixr provides the same analysis plots available in the ConsensusClusterPlus package. We recommend the visualisation of the consensus matrices, the consensus CDF plot and the item-consensus plots to determine the optimal number of clusters. In short, a good cluster solution should show a clear separation of the clusters in the consensus matrix and its line in the consensus CDF plot should show a binary separation (steep ascent at 0, then flat and another steep ascent towards 1). Additionally, the cluster separation should be visible in the item-consensus plot, meaning that all observations in one cluster should have a high average consensus value with the other members of this cluster and a low consensus value with members of different clusters. The plots can be produced by calling the plot function on an lcc object generated by the longmixr package. As it can be the case that the flexmix models find less clusters than specified, the median number of found clusters is also mentioned in the title of the plots.

Plot the diagnostics plots:

```
plot(model, which_plots = "consensusmatrix_legend")
```

```
plot(model, which_plots = "consensusmatrix_2")
```

```
plot(model, which_plots = "consensusmatrix_3")
```

```
plot(model, which_plots = "consensusmatrix_4")
```

```
plot(model, which_plots = "consensusmatrix_5")
```

```
plot(model, which_plots = "consensusmatrix_6")
```

```
plot(model, which_plots = "CDF")
```

```
plot(model, which_plots = "item_consensus", n_item_consens = 5)
```

Based on the consensus matrices, the consensus CDF and the item-consensus plots, we determined 2 clusters as the optimal cluster solution. The clusters differed significantly in size. 82.9% (n=63) of the sample was assigned to cluster 1. All subsequent visualizations are based on the results from 2 clusters.

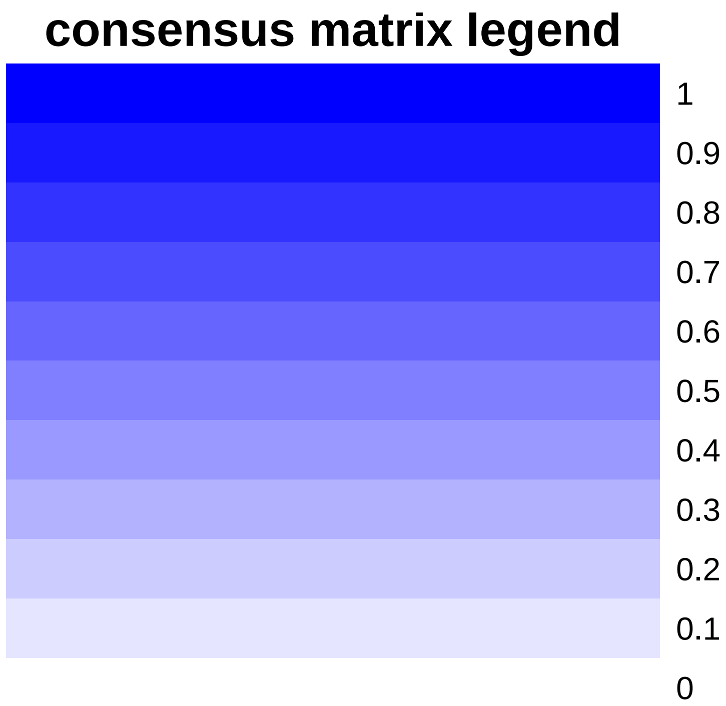

Figure S8: Legend for consensus matrix plots.

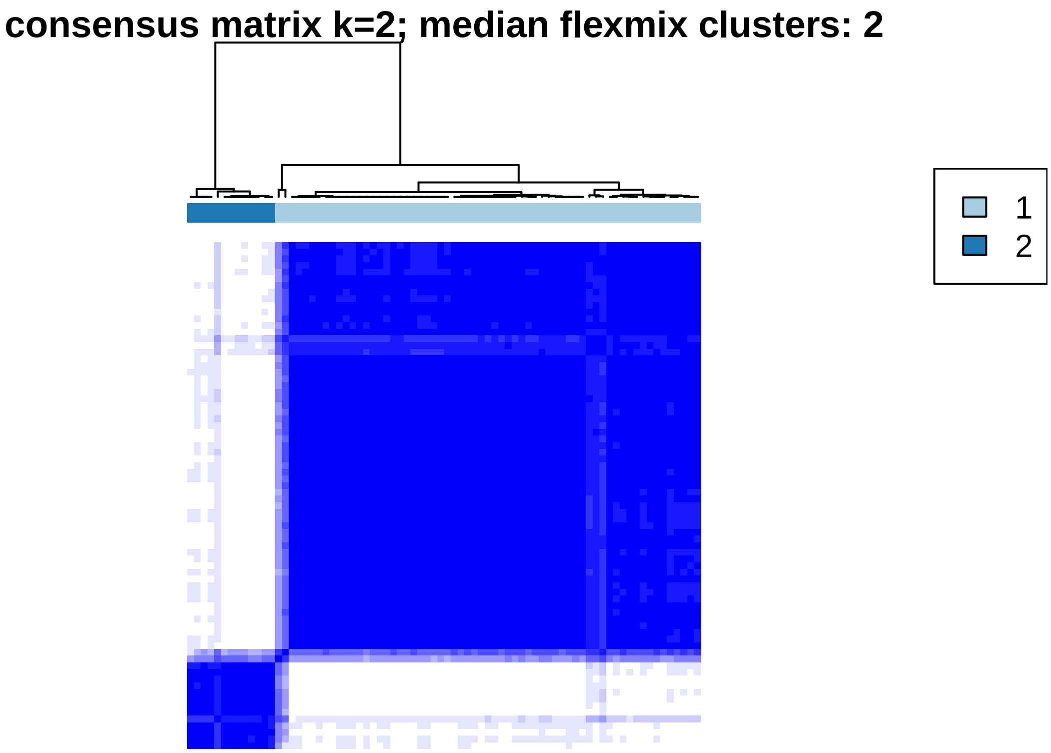

Figure S9: Consensus matrix for a 2 cluster solution.

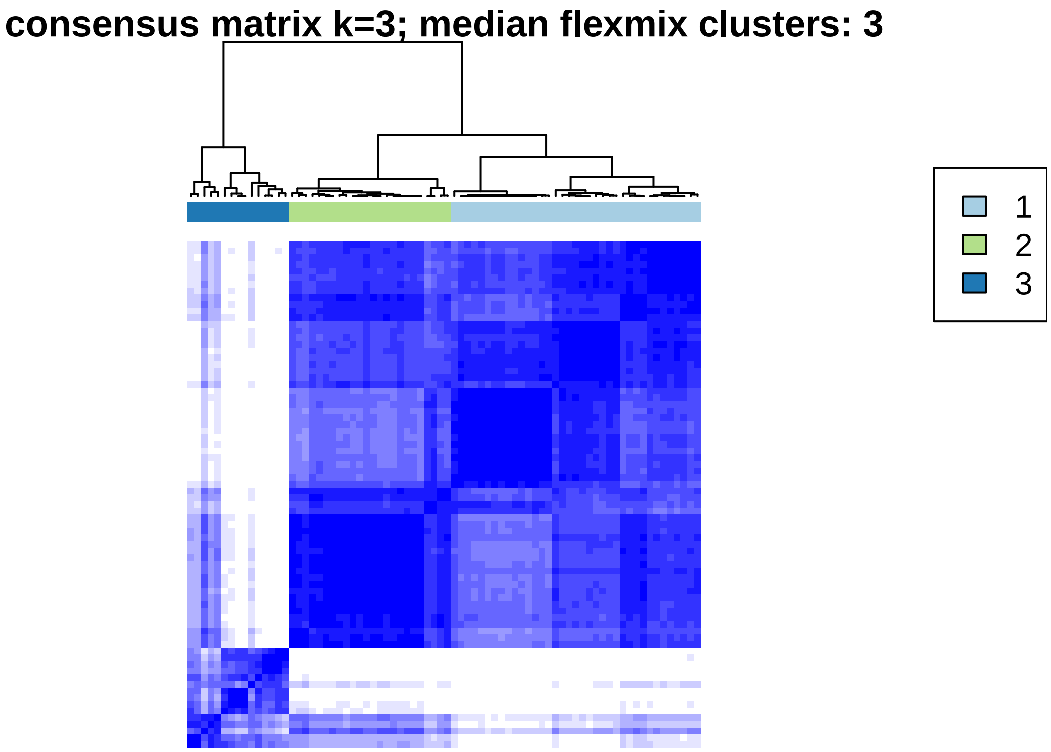

Figure S10: Consensus matrix for a 3 cluster solution.

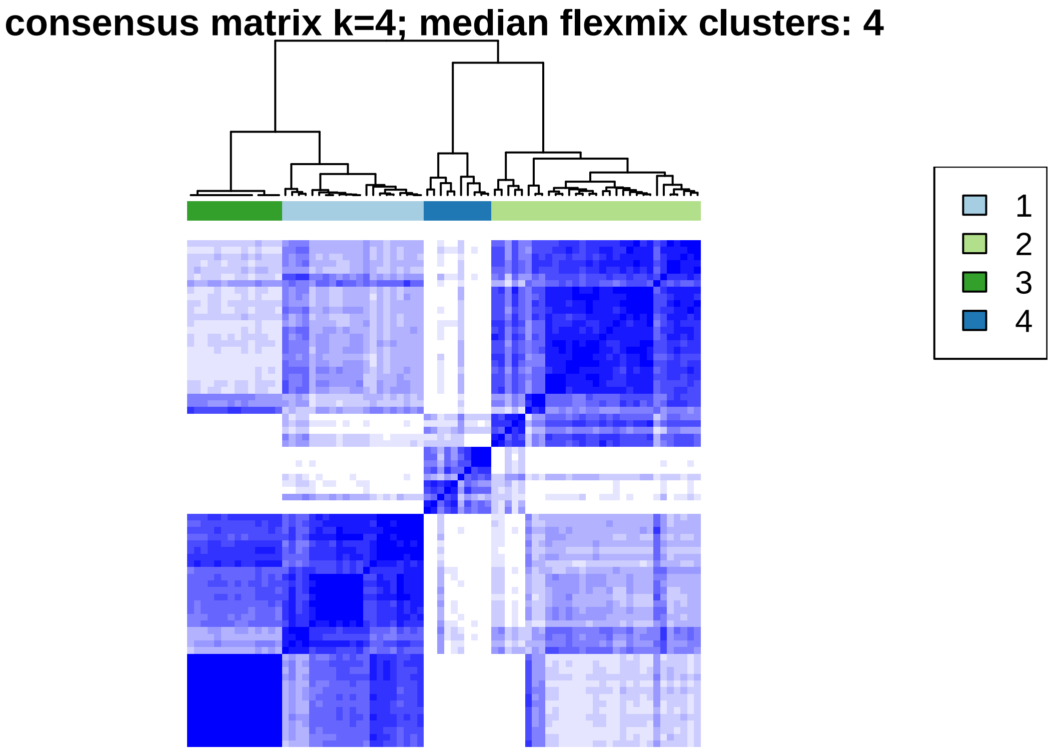

Figure S11: Consensus matrix for a 4 cluster solution.

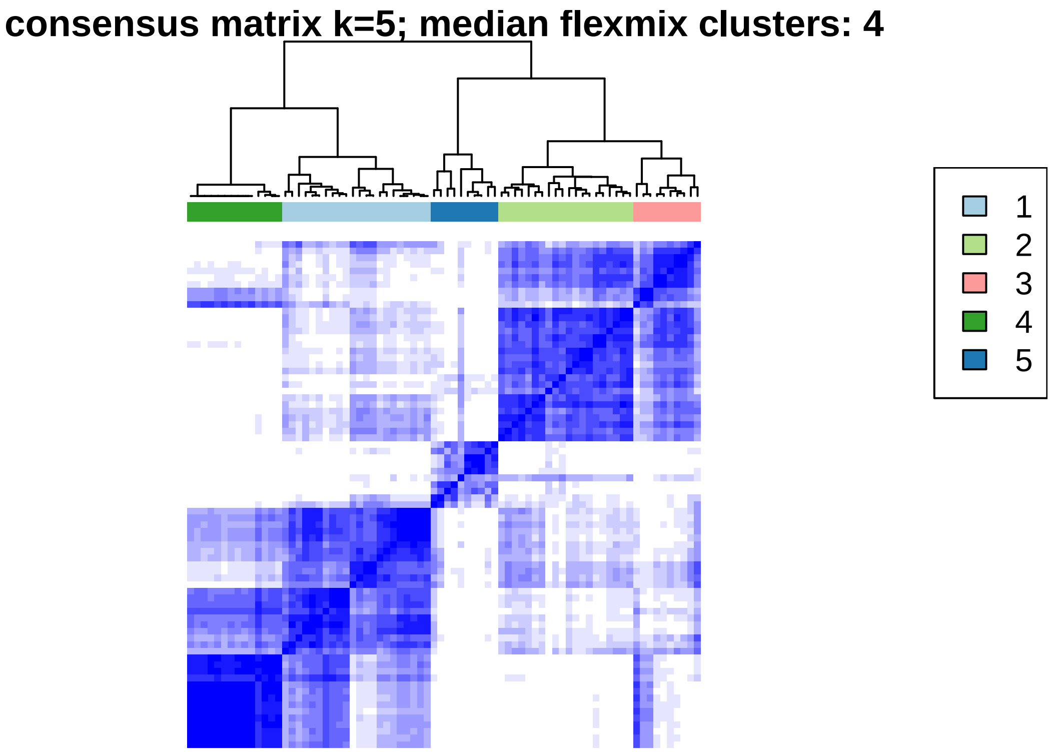

Figure S12: Consensus matrix for a 5 cluster solution.

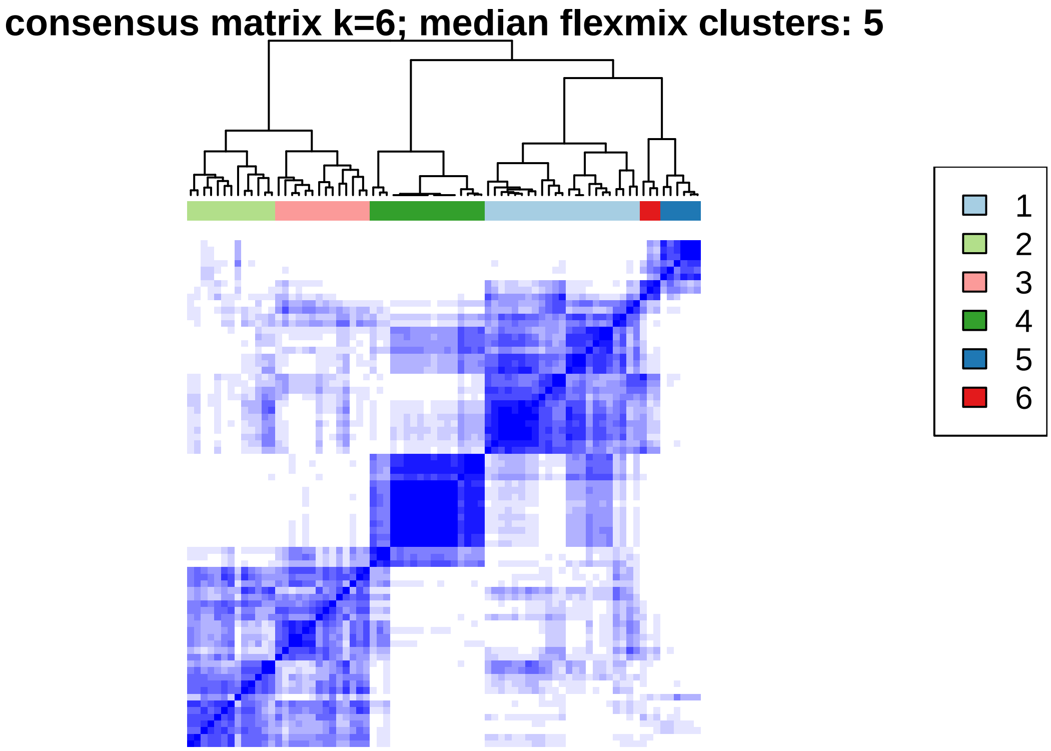

Figure S13: Consensus matrix for a 6 cluster solution.

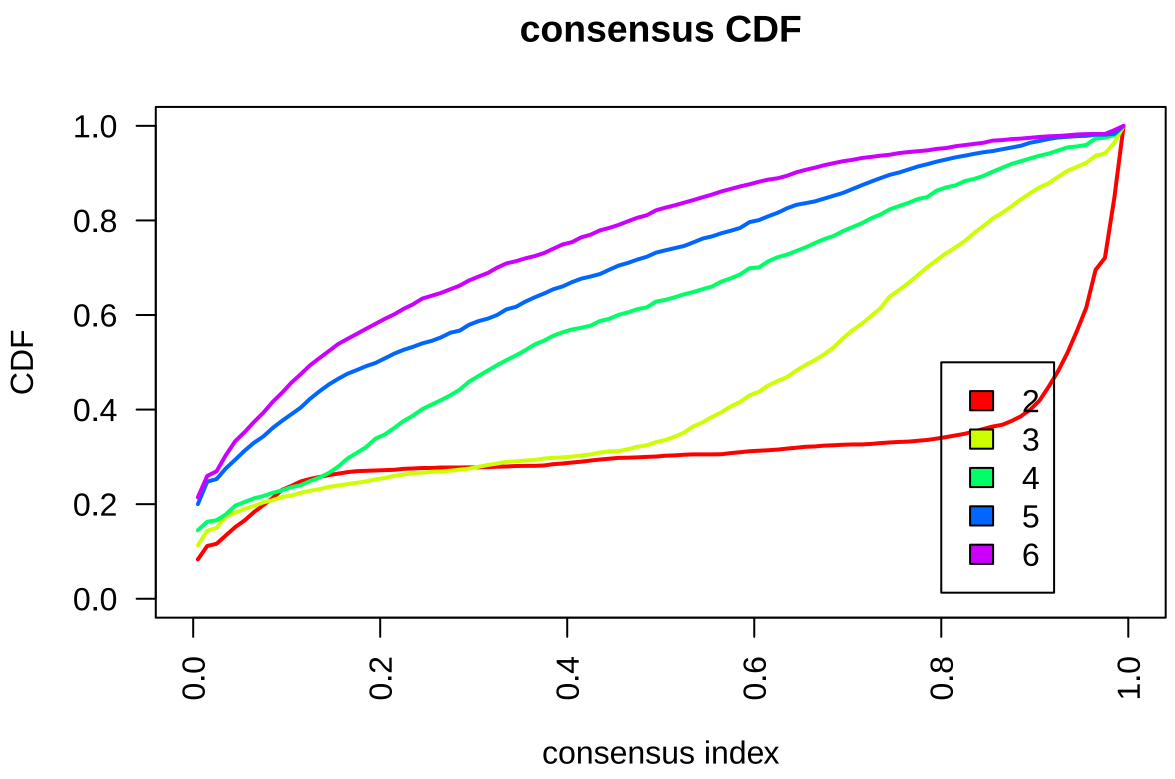

Figure S14: CDF plot.

3.2.5.2 Visualisation of the results Spaghetti plots are used to visualize the results of the longitudinal clustering. They show the trajectories of the components (from the dimension reduction) over the time points for the different clusters. To visualize how the original categorical variables change over time for the different clusters, we use alluvial plots based on the R package ggalluvial (Brunson, 2020; Brunson and Read, 2023). longmixr contains built-in plotting functions for these types of plots. For further interpretation of the clusters, they can be compared regarding time-invariant data not used for the clustering. Results from these comparisons are visualized with bar charts or box plots depending on the data type. The same applies to the results of cross-sectional clustering.

3.2.5.2.1 Variables used for the clustering Have a look at the distribution of the variables that resulted from the data set with reduced dimensions and were used in the clustering.

```
all_cluster_assignments <- get_clusters(model)
```

Residuals of components after age regression

In bold, the mean is shown, the transparent area depicts plus/minus one standard deviation around the mean.

```
plot_spaghetti(
  model = model,
  data = cluster_data_resid,
  variable_names = paste0("panss_", 1:5, "_resid"),
  time_variable = "visit",
  number_of_clusters = 2
)
```

3.2.5.2.2 Original variables Most of the original variables are categorical variables. For a better visualization of the change over time, we use alluvial plots.

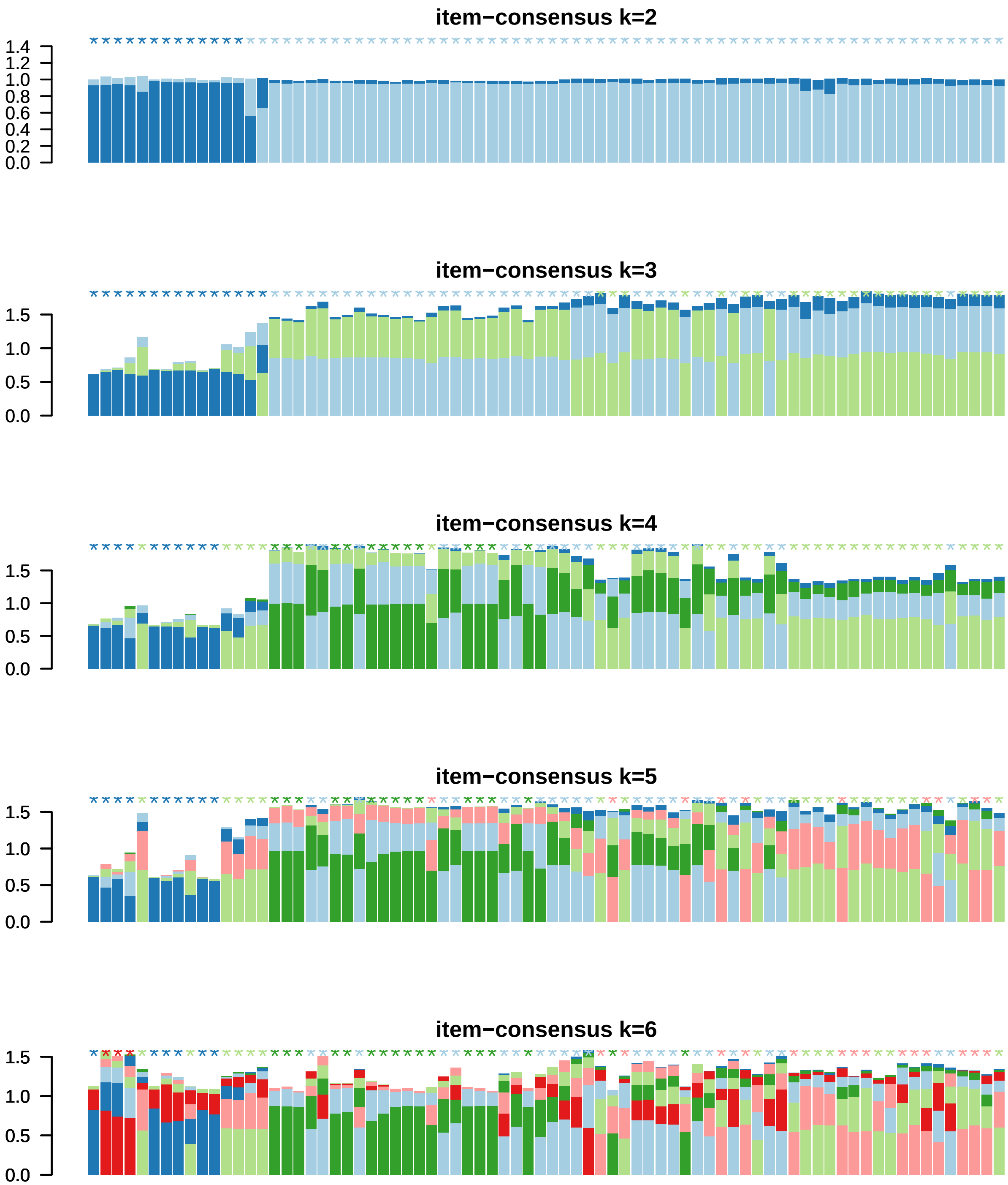

Figure S15: Item consensus plots.

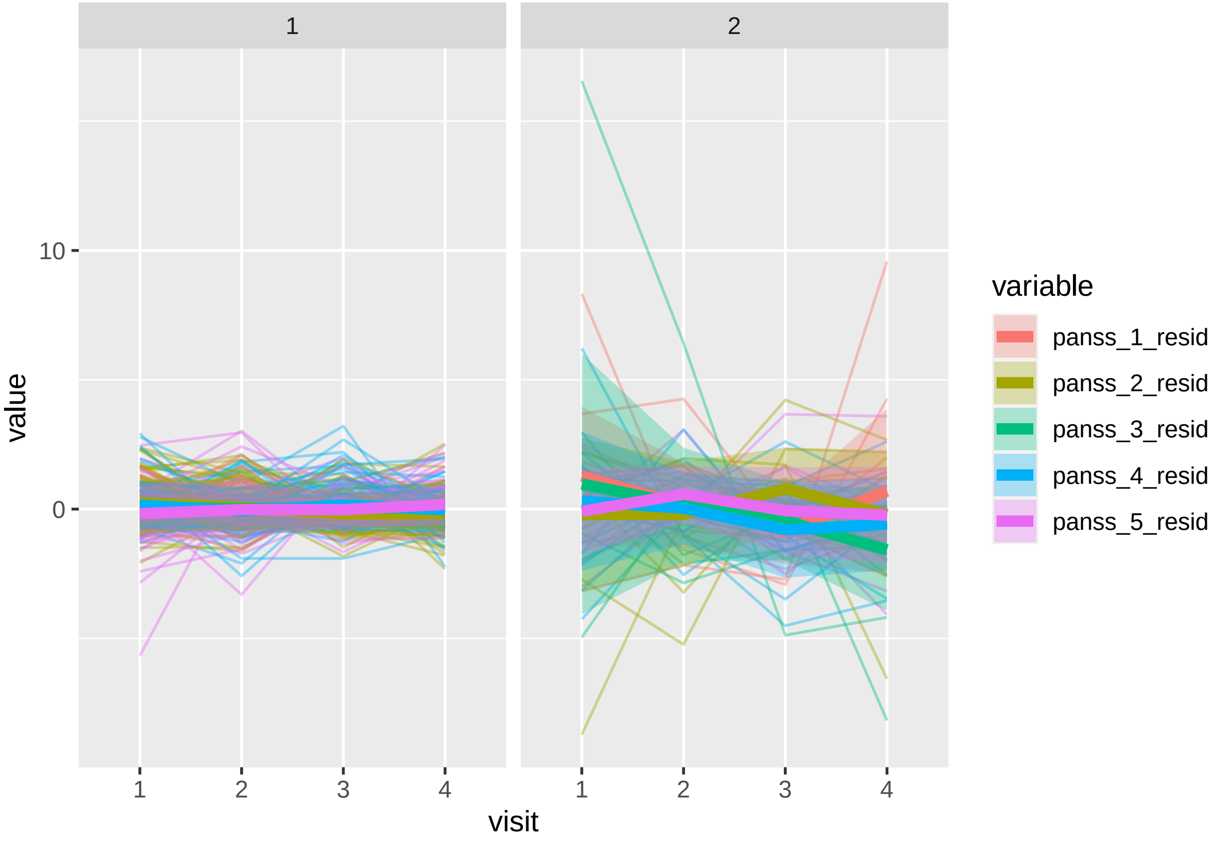

Figure S16: Distribution of residuals of PANSS components across clusters.

```r
plot_data <- data %>%
  rename(patient_id = v1_id)
```

panss variables:

```r
plot_alluvial(model = model, data = plot_data, time_variable = "visit",
              variable_name = "panss_n1")
```

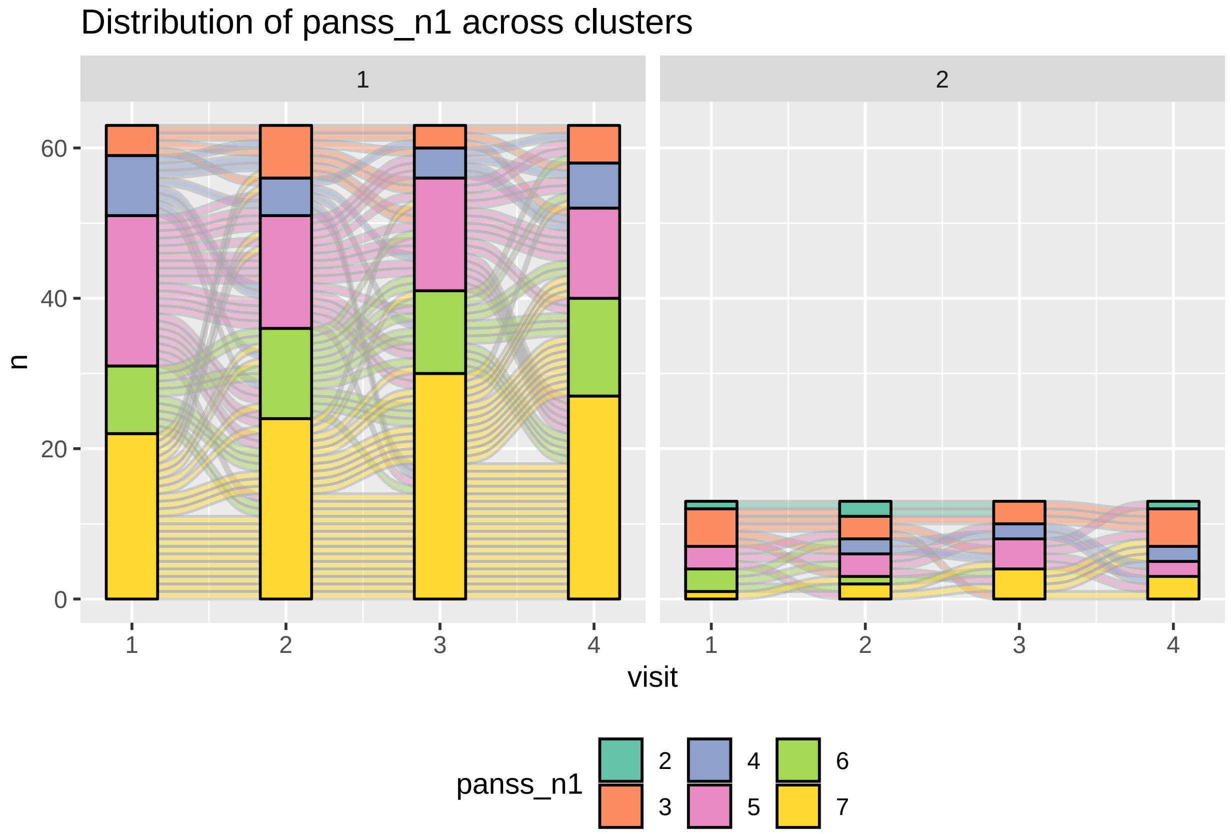

Figure S17: Distribution of original negative symptom 1 of PANSS across clusters. Higher values correspond to lower symptom burden.

```r
plot_alluvial(model = model, data = plot_data, time_variable = "visit",
              variable_name = "panss_n2")
```

```r
plot_alluvial(model = model, data = plot_data, time_variable = "visit",
              variable_name = "panss_n3")
```

```r
plot_alluvial(model = model, data = plot_data, time_variable = "visit",
              variable_name = "panss_n4")
```

```r
plot_alluvial(model = model, data = plot_data, time_variable = "visit",
              variable_name = "panss_n5")
```

```r
plot_alluvial(model = model, data = plot_data, time_variable = "visit",
              variable_name = "panss_n6")
```

```r
plot_alluvial(model = model, data = plot_data, time_variable = "visit",
              variable_name = "panss_n7")
```

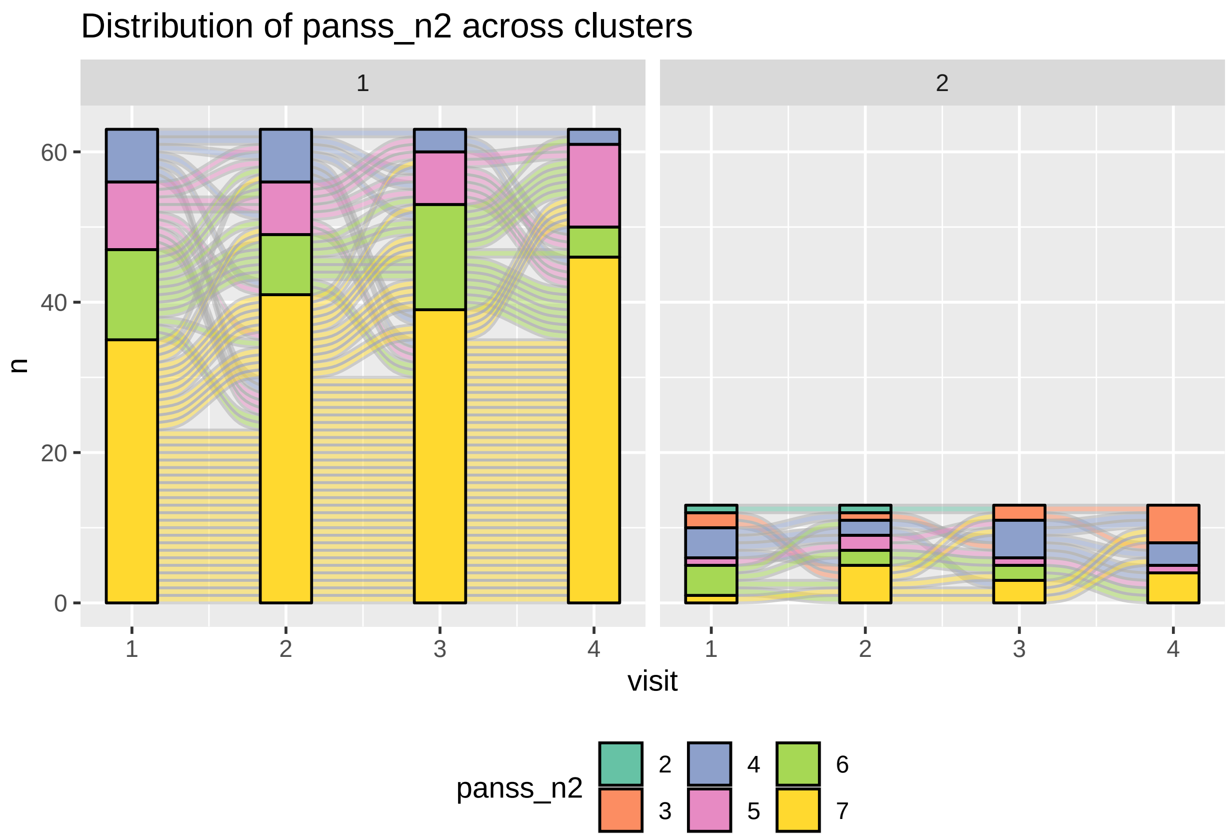

Figure S18: Distribution of original negative symptom 2 of PANSS across clusters. Higher values correspond to lower symptom burden.

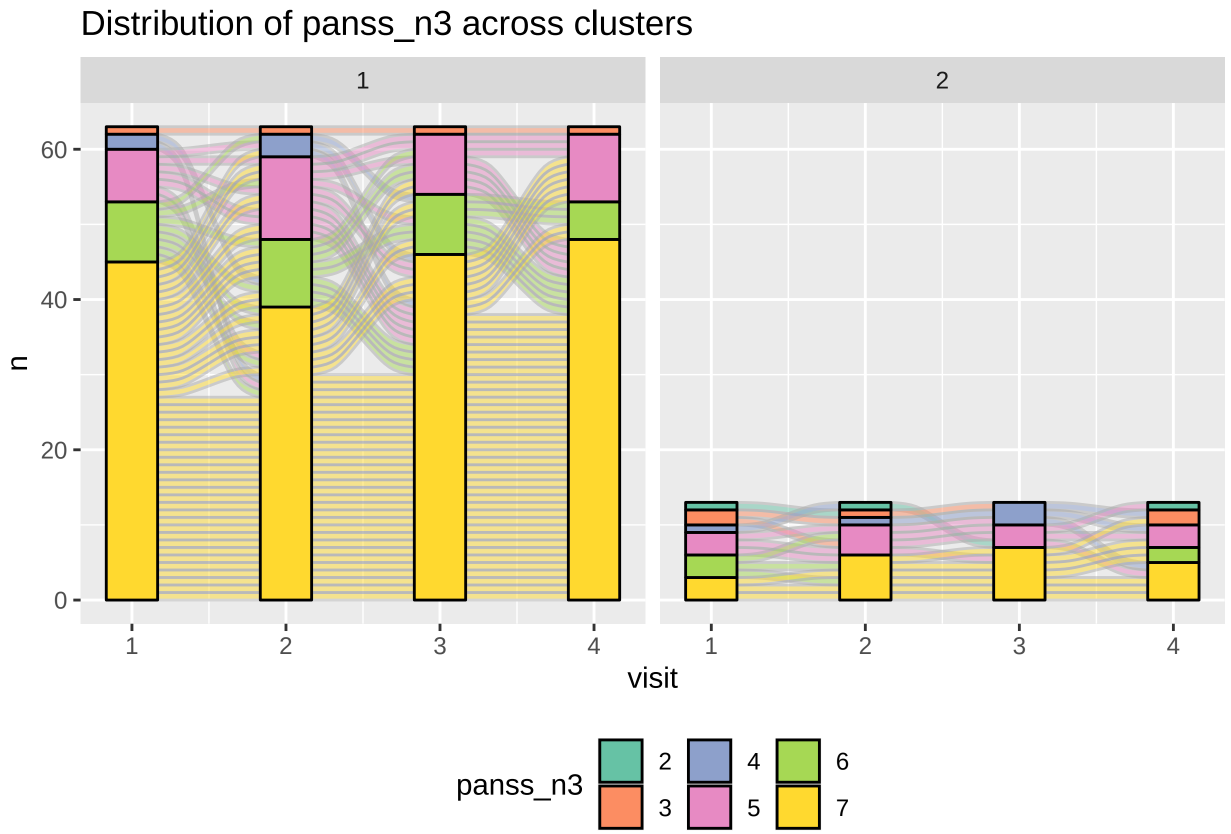

Figure S19: Distribution of original negative symptom 3 of PANSS across clusters. Higher values correspond to lower symptom burden.

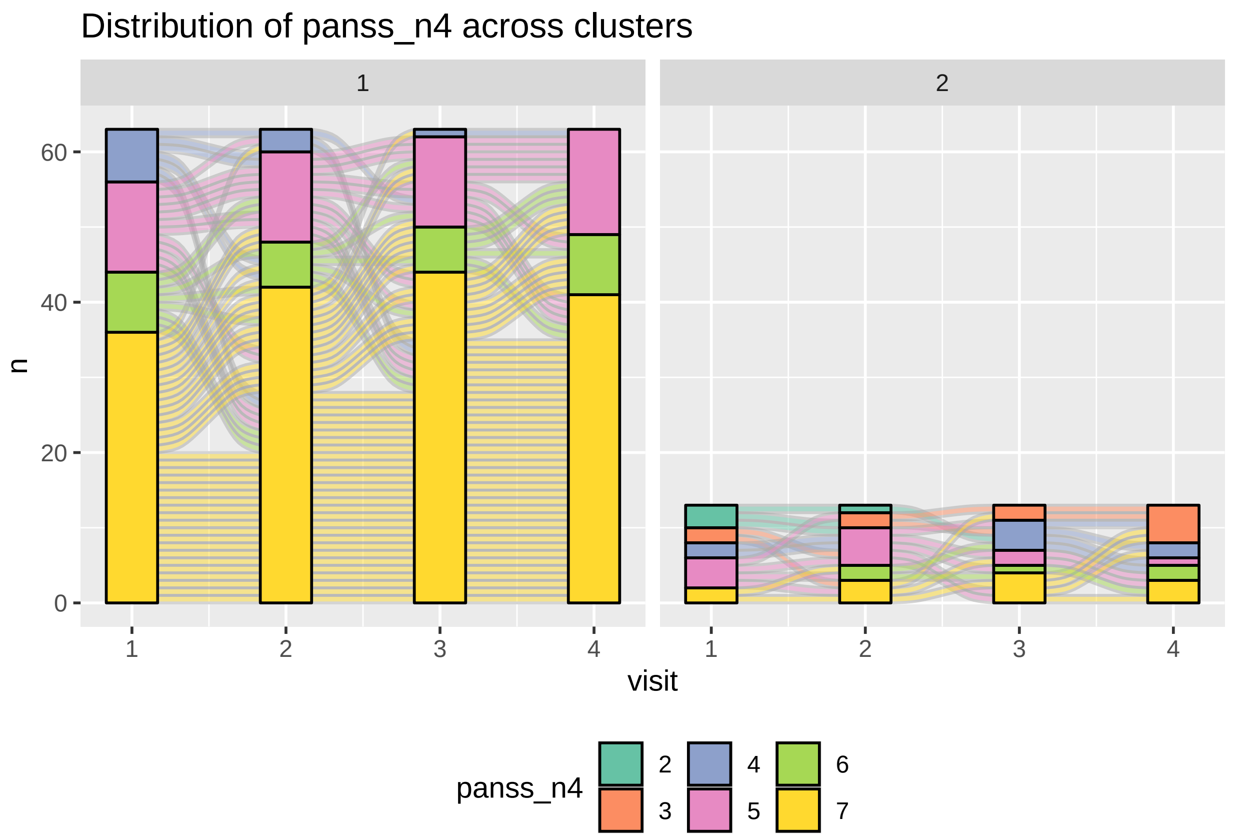

Figure S20: Distribution of original negative symptom 4 of PANSS across clusters. Higher values correspond to lower symptom burden.

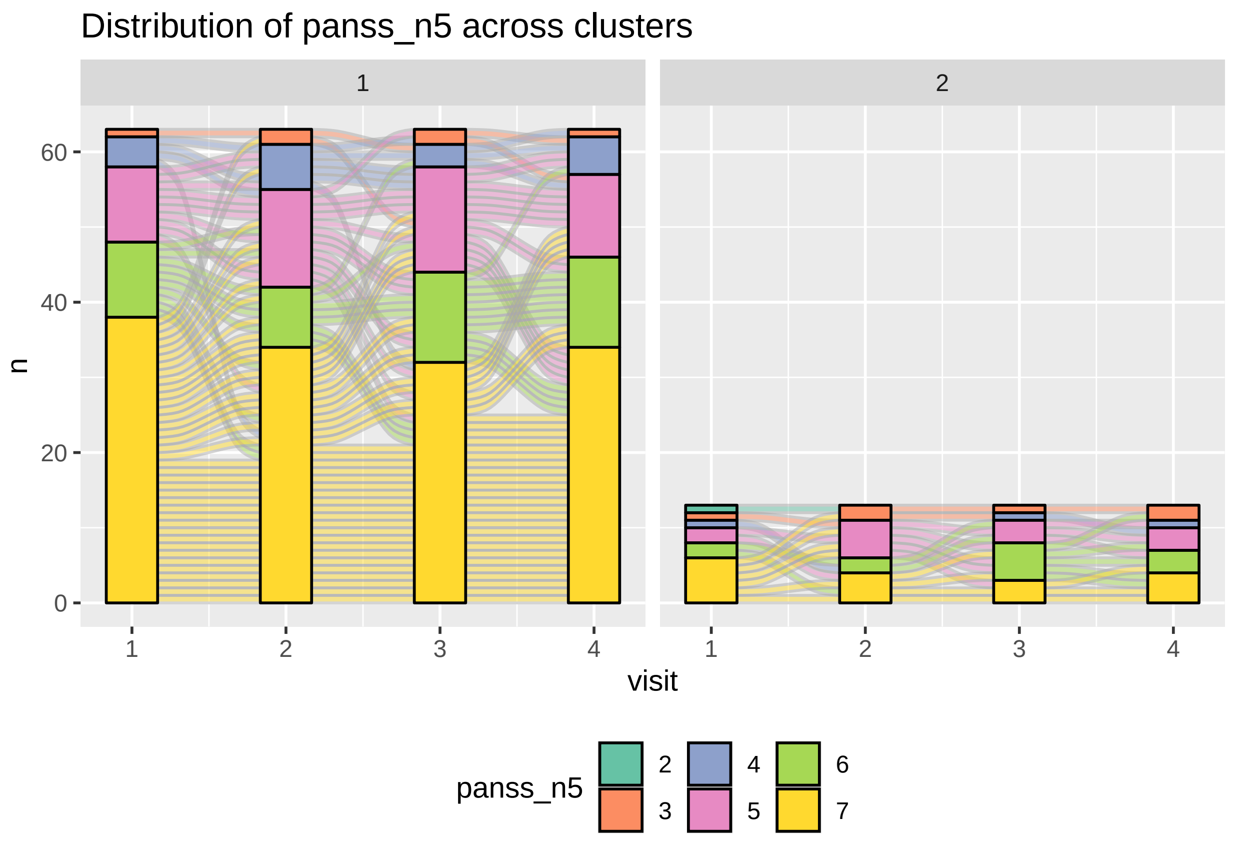

Figure S21: Distribution of original negative symptom 5 of PANSS across clusters. Higher values correspond to lower symptom burden.

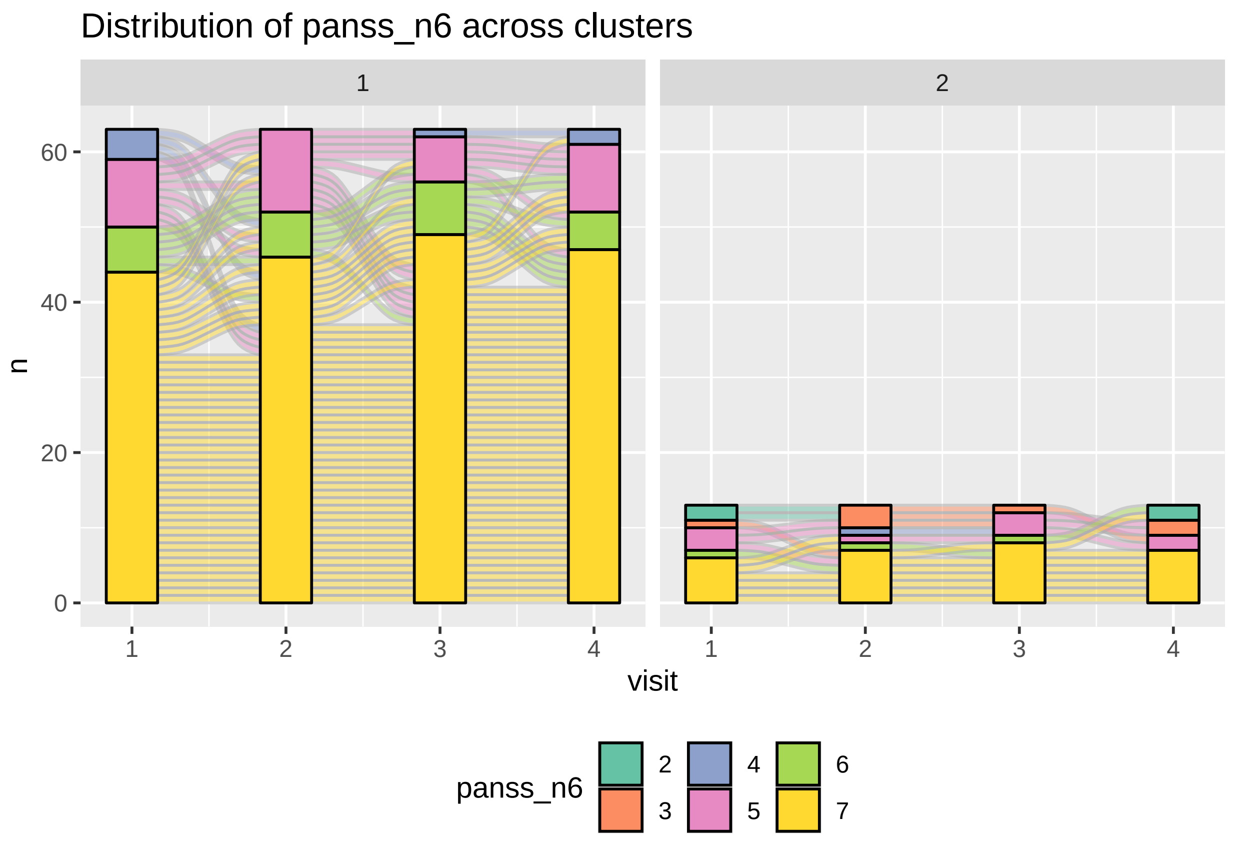

Figure S22: Distribution of original negative symptom 6 of PANSS across clusters. Higher values correspond to lower symptom burden.

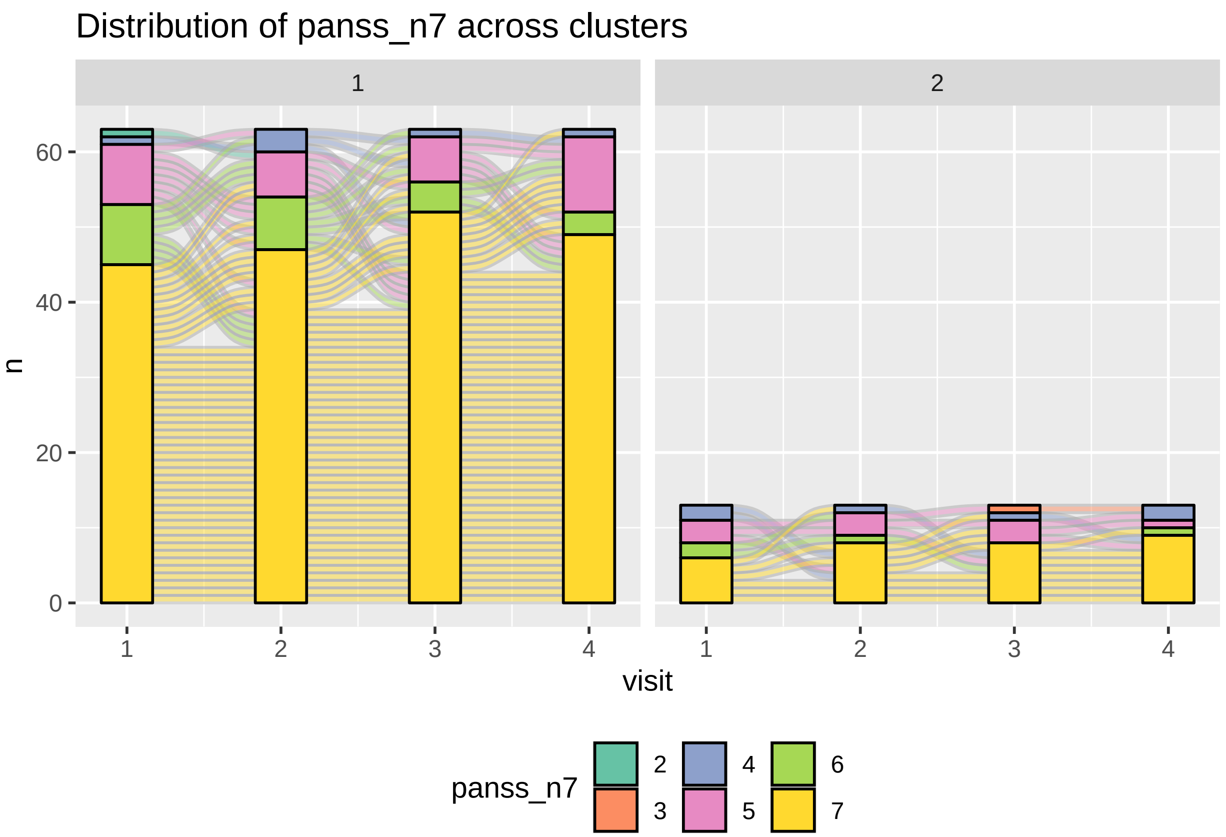

Figure S23: Distribution of original negative symptom 7 of PANSS across clusters. Higher values correspond to lower symptom burden.

3.2.5.3 Optimal clustering solution

The investigation of the diagnostic plots shows that in this use case a two cluster solution is optimal (in the following referred to as C1 and C2), see S8 - S15 as the consensus matrix and item consensus plot for two clusters are best separated and the consensus CDF for two clusters shows a steep ascend at 0, then a flat line and again a steep ascend at 1.

3.2.5.4 Characterization of the clusters

Most subjects (n=63) are assigned to C1. C1 is characterized by stable average scores over the 18 months study period on all dimensions representing psychopathological symptoms, i.e. all dimensions derived from IDS-C_{30} (not shown), PANSS Negative Scale and BDI-II (not shown). In comparison, patients in C2 show a higher variability both overall as well as in regards to changes over time, even though the mean level of symptoms is comparable between the clusters. Interestingly, in C2 the original sum scores of depressive symptoms rated on both a self rating scale (BDI-II) and by a trained interviewer (IDS-C_{30}) decreased at visits 2 and 3 compared to baseline but rose back up at visit 4, while the BDI-II and IDS-C_{30} sum scores in C1 stayed relatively constant over time (not shown). Subjects in C1 improve on average over time on the first dimension of functioning, while their scores on the second dimension of functioning dip at visit 2 and then stabilize again at visits 3 and 4 (not shown). Subjects in C2 on the other hand peak at visit 3 on the first dimension, while their scores on the second dimension slightly decrease from visit 1 to visit 3 and then stabilize at visit 4 (not shown).

For a further characterization of the clusters, we compared them on variables that were not considered in the clustering process, namely age, sex and quality of life (QoL) in the global domain. QoL was assessed with the World Health Organization Quality-of-Life Scale (WHOQOL-BREF). Subjects in C1 (mean age (years) = 42.4, SD = 11.3) were older compared to subjects in C2 (mean age (years) = 38.8, SD = 8.5), but the difference was not significant, t(21.65) = 1.3, p = .197. The proportion of female subjects was 36.5% in C1 and 30.8% in C2. A chi-square test of independence was performed to examine the relation between gender and cluster assignment. The relation between these variables was not significant, χ^2 (1, N = 76) = 0.0057, p = 0.94.

3.2.5.4.1 Quality of life across clusters

The longitudinal association of QoL with the clusters is assessed with linear mixed models.

```
data_qol <- psycrs3.0_lng %>%
  select(v1_id, v1_sex, age, bmi, whoqol_dom_env, whoqol_dom_glob,
         whoqol_dom_phys, whoqol_dom_psy, whoqol_dom_soc, visit) %>%
  rename(patient_id = v1_id) %>%
  right_join(all_cluster_assignments, by = "patient_id") %>%
  mutate(assignment_num_clus_2 = as.factor(assignment_num_clus_2),
         visit = as.factor(visit))

print_summary_table <- function(data, caption) {
  print_data <- data$tTable %>%
    as.data.frame()
  rownames(print_data) <- NULL

  print_data <- print_data %>%
    mutate(Value = format(Value, digits = 2, justify = "right"),
           Std.Error = format(Std.Error, digits = 2, justify = "right"),
           `t-value` = format(`t-value`, digits = 2),
           `p-value` = round(`p-value`, digits = 3),
           `p-value` = format(`p-value`, digits = 3),
           `p-value` = if_else(`p-value` < 0.001, "<0.001", as.character(`p-value`)),
           type = c(rep("Main effects", 5), rep("Interaction effects", 3)),
           predictor = c("Intercept", "Visit 2", "Visit 3", "Visit 4",
                         "Cluster 2", "Visit 2 x Cluster 2",
                         "Visit 3 x Cluster 2", "Visit 4 x Cluster 2")) %>%
    relocate(type, predictor)
```

Table S2: Longitudinal comparison of global quality of life between clusters.

		Value	Std.Error	DF	t-value	p-value
**Main effects**						
	**Intercept**	13.48	0.44	219	30.94	<0.001
	**Visit 2**	0.33	0.36	219	0.91	0.364
	**Visit 3**	0.33	0.36	219	0.91	0.364
	**Visit 4**	0.32	0.36	219	0.90	0.371
	**Cluster 2**	-1.94	1.05	74	-1.85	0.068
**Interaction effects**						
	**Visit 2 x Cluster 2**	-2.09	0.89	219	-2.36	0.019
	**Visit 3 x Cluster 2**	-1.10	0.87	219	-1.27	0.207
	**Visit 4 x Cluster 2**	-1.55	0.87	219	-1.79	0.074

Note:

Std.Error = Standard Error; DF = degrees of freedom

```
  colnames(print_data)[2] <- ""

  print_data %>%
    kbl(align = c("l", "l", "r", "r", "r", "r", "r"),
        booktabs = TRUE,
        caption = caption) %>%
    column_spec(2, bold = TRUE) %>%
    collapse_rows(columns = 1:2, row_group_label_position = "stack",
                  latex_hline = "none") %>%
    footnote(general = linebreak("Std.Error = Standard Error; DF = degrees of freedom"))
}
```

```
data_qol %>% filter(is.na(whoqol_dom_glob)) %>% distinct(patient_id) %>% nrow()
```

```
## [1] 3
```

```
# whoqol_dom_glob has 3 missing values, remove these
model_data <- data_qol %>%
  filter(!is.na(whoqol_dom_glob))

model_2 <- lme(whoqol_dom_glob ~ visit*assignment_num_clus_2,
               random = ~1|patient_id,
                data = model_data)
sum_data_2 <- summary(model_2)
```

The results of the longitudinal comparisons of QoL data between the clusters (determined by linear mixed models) are presented in Table S2.

```
print_summary_table(sum_data_2,
                    caption = "Longitudinal comparison of global quality of life between clusters.")
```

Interestingly, in line with the psychopathological assessments used in the clustering, patients in C1 show the trend of a higher QoL in the global QoL domain. The global QoL does not show a main effect of time. However, a significant interaction between cluster membership and time can be found in this domain. The QoL scores decrease at visit 2 compared to baseline, but only in C2.

In this comparatively homogeneous group of patients, the cluster algorithm was able to select patients with a

higher variability over time. This might be an interesting group for further investigations.

References

Brunson,J.C. (2020) ggalluvial: Layered grammar for alluvial plots. *Journal of Open Source Software*, **5**, 2017.

Brunson,J.C. and Read,Q.D. (2023) Ggalluvial: Alluvial plots in 'ggplot2' The Comprehensive R Archive Network.

Budde,M. *et al.* (2019) A longitudinal approach to biological psychiatric research: The PsyCourse study. *American Journal of Medical Genetics Part B: Neuropsychiatric Genetics*, **180**, 89–102.

D'Orazio,M. (2022) StatMatch: Statistical matching or data fusion The Comprehensive R Archive Network.

Grün,B. and Leisch,F. (2008) FlexMix version 2: Finite mixtures with concomitant variables and varying and constant parameters. *Journal of Statistical Software*, **28**, 1–35.

Grün,B. and Leisch,F. (2020) Flexmix: Flexible mixture modeling The Comprehensive R Archive Network.

Honaker,J. *et al.* (2011) Amelia II: A program for missing data. *Journal of statistical software*, **45**, 1–47.

Honaker,J. *et al.* (2018) Amelia: A program for missing data The Comprehensive R Archive Network.

Husson,F. *et al.* (2020) FactoMineR: Multivariate exploratory data analysis and data mining The Comprehensive R Archive Network.

Kahn,R.S. *et al.* (2015) Schizophrenia. *Nature Reviews Disease Primers*, **1**, 15067.

Laliberté,E. *et al.* (2014) FD: Measuring functional diversity from multiple traits, and other tools for functional ecology The Comprehensive R Archive Network.

Laliberté,E. and Legendre,P. (2010) A distance-based framework for measuring functional diversity from multiple traits. *Ecology*, **91**, 299–305.

Lê,S. *et al.* (2008) FactoMineR: An r package for multivariate analysis. *Journal of statistical software*, **25**, 1–18.

Leisch,F. (2004) Flexmix: A general framework for finite mixture models and latent glass regression in r.

Maechler,M. *et al.* (2021) Cluster: Cluster analysis basics and extensions The Comprehensive R Archive Network.

Miyamoto,S. *et al.* (2015) Ward method of hierarchical clustering for non-euclidean similarity measures. In, *2015 7th international conference of soft computing and pattern recognition (SoCPaR)*. IEEE, pp. 60–63.

Monti,S. *et al.* (2003) Consensus clustering: A resampling-based method for class discovery and visualization of gene expression microarray data. *Machine learning*, **52**, 91–118.

Wilkerson,M.D. and Hayes,D.N. (2010) ConsensusClusterPlus: A class discovery tool with confidence assessments and item tracking. *Bioinformatics*, **26**, 1572–1573.

Wilkerson,M.D. and Hayes,D.N. (2020) ConsensusClusterPlus: A class discovery tool with confidence assessments and item tracking Bioconductor.
